# Segregation between breeds and local breed proportions in genetic and genomic models for crossbreds

**DOI:** 10.1186/s12711-023-00810-5

**Published:** 2023-07-05

**Authors:** Jón H. Eiríksson, Guosheng Su, Ismo Strandén, Ole F. Christensen

**Affiliations:** 1 Center for Quantitative Genetics and Genomics, Aarhus University, 8000 Aarhus C, Denmark; 2 Present Address: Faculty of Agricultural Sciences, Agricultural University of Iceland, 311 Borgarnes, Iceland; 3 Natural Resources Institute Finland (Luke), 31600 Jokioinen, Finland

## Abstract

**Background:**

The breeding value of a crossbred individual can be expressed as the sum of the contributions from each of the contributing pure breeds. In theory, the breeding value should account for segregation between breeds, which results from the difference in the mean contribution of loci between breeds, which in turn is caused by differences in allele frequencies between breeds. However, with multiple generations of crossbreeding, how to account for breed segregation in genomic models that split the breeding value of crossbreds based on breed origin of alleles (BOA) is not known. Furthermore, local breed proportions (LBP) have been modelled based on BOA and is a concept related to breed segregation. The objectives of this study were to explore the theoretical background of the effect of LBP and how it relates to breed segregation and to investigate how to incorporate breed segregation (co)variance in genomic BOA models.

**Results:**

We showed that LBP effects result from the difference in the mean contribution of loci between breeds in an additive genetic model, i.e. breed segregation effects. We found that the (co)variance structure for BS effects in genomic BOA models does not lead to relationship matrices that are positive semi-definite in all cases. However, by setting one breed as a reference breed, a valid (co)variance structure can be constructed by including LBP effects for all other breeds and assuming them to be correlated. We successfully estimated variance components for a genomic BOA model with LBP effects in a simulated example.

**Conclusions:**

Breed segregation effects and LBP effects are two alternative ways to account for the contribution of differences in the mean effects of loci between breeds. When the covariance between LBP effects across breeds is included in the model, a valid (co)variance structure for LBP effects can be constructed by setting one breed as reference breed and fitting an LBP effect for each of the other breeds.

**Supplementary Information:**

The online version contains supplementary material available at 10.1186/s12711-023-00810-5.

## Background

Estimation of breeding values relies on additive genetic (co)variances between individuals. These (co)variances are usually derived from two types of information, i.e. pedigree or genotypes, where the latter are typically based on genotypes for genome-wide single nucleotide polymorphisms (SNPs) [1]. These two sources of information can also be combined in single-step genomic models [2, 3]. The general methods for estimating these (co)variances have been developed for purebred populations and thus modifications are needed for genetic evaluation of crossbred populations [4, 5].

For crossbred populations, other than the first generation of crossbreeding (F1), the additive genetic (co)variances include both contributions from each of the contributing pure breeds and contributions from segregation between breeds (breed segregation, BS) [4]. García-Cortés and Toro [6] presented a pedigree-based model for the genetic evaluation of crossbred populations in which the additive genetic (co)variances for crossbreds of any breed combination were partitioned into breed-specific terms and BS terms. In their model [6], each breed-specific and BS variance–covariance term is the product of a partial genetic variance component and a partial genetic relationship matrix. However, the inclusion of genomic information in that model is not straightforward because of the separate BS terms for each pair of breeds.

For genomic relationships, Strandén and Mäntysaari [7] presented a random regression approximation of the García-Cortés and Toro [6] model by partitioning the breeding value by breed proportions. Other studies suggested that the breed origin of alleles (BOA) should be accounted for in genomic evaluations for crossbreds [5, 8–10]. For genomic BOA models, the breed origins of the marker alleles are traced and their effects are allowed to depend on breed origin to account for breed differences in marker allele effects, e.g. due to differences in the linkage disequilibrium between markers and quantitative trait loci (QTL) and differences in the genetic background between breeds [8, 9]. Thus, the partitioning of breeding values into breed-specific effects is more accurate in BOA models than when the partitioning is based on the breed proportions alone. Christensen et al. [5] considered only the first generation of crossbreeding, for which BS is not present, while Ibánẽz-Escriche et al. [8] ignored BS for three-way and four-way crossbreeding, and Karaman et al. [9] and Eiríksson et al. [10] ignored BS in genomic BOA models for rotational crossbreeding systems. For a three-way terminal crossbreeding system, Christensen et al. [11] presented a single-step genomic model by partitioning the breeding value into breed-specific terms according to BOA and a BS term for segregation between the two maternal breeds. In that paper [11], a BS partial relationship for the segregation between the two maternal breeds was constructed based on information on BOA. For a population of inbred maize lines with lines from two genetic groups and admixed lines, Rio et al. [12] presented a genomic model with group-specific effects and a BS term. Recently, Aase et al. [13] presented an extension of the model of Rio et al. [12], adapted for an admixture of multiple groups in wild animal populations but ignored the BS term, although formulas for BS covariances were given in their Supplementary material [13], but without fully considering the properties of the proposed (co)variance structure. We are not aware of any published studies that explain how to construct BS partial relationships in genomic BOA models for crossbred populations with any breed composition.

A concept that accounts for genetic effects that are not accounted for by the breed-specific genetic effects, similar to BS, is local breed proportion (LBP) effects [14]. For crossbred dairy cattle, Eiríksson et al. [14] fitted LBP effects with random regressions on the proportion of alleles assigned to each breed origin within chromosome segments or for individual SNPs. They reported a low but statistically significant estimate of variance related to the LBP effects for milk production traits. Similarly, Bolormaa et al. [15] investigated the effect of local zebu or taurine ancestry of chromosome segments on phenotypes in Australian composite beef cattle and found that ancestry at some positions affected the studied traits. The effects of LBP, or of local ancestry, have similarities with BS but are defined for individual breeds rather than between pairs of breeds. However, the theoretical background of LBP and the exact relationship between LBP and BS is unclear.

Therefore, the objectives of this study were: (1) to derive and present the theoretical background of the effects of LBP in genetic models and how they are related to BS effects, (2) to investigate how BS effects can be included in genomic BOA models, and (3) to present variance structure that can be included in genetic and genomic models for crossbred populations with any breed composition to account for BS variance.

## Methods

We start this section by reviewing the theory behind partitioning the breeding value into breed-specific terms and BS or LBP terms in genetic models. Second, we review the construction of breed-specific partial relationship matrices for these models. Third, we show that the (co)variance structure for BS effects based on BOA does not lead to relationship matrices that have a quadratic form. Finally, we present an alternative method for the inclusion of LBP effects in both pedigree-based and genomic BOA models, which accounts for BS and results in valid relationship matrices and variance components.

### Theory

We start by reviewing the theory behind models for partitioning the additive genetic value of crossbreds into breed-specific terms [6]. We specify the additive genetic value of individual $i$ as follows (inspired by Lo et al. [4] and the Appendix of Christensen et al. [11]):

1 $${g}_{i}=\sum \limits_{j=1}^{m}\left({\alpha }_{{s}_{i}^{j}}+{\alpha }_{{d}_{i}^{j}}\right),$$

where ${\alpha }_{{s}_{i}^{j}}$ is the additive effect for the individual’s paternal allele at locus $j$, ${\alpha }_{{d}_{i}^{j}}$ is the additive effect for its maternal allele at locus $j$, and $m$ is the number of loci. The effects, ${\alpha }_{{s}_{i}^{j}}$ and ${\alpha }_{{d}_{i}^{j}}$, depend on BOA. We introduce the terms ${r}_{{s}_{i}^{j}}^{b}$ and ${r}_{{d}_{i}^{j}}^{b}$, which indicate whether the maternal and paternal alleles, respectively, at locus $j$ originate from breed $b$, with ${r}_{{s}_{i}^{j}}^{b}=1$ $({r}_{{d}_{i}^{j}}^{b}=1)$ when the paternal (maternal) allele is from breed $b$ and zero otherwise. We define the effect of local ancestry as the mean additive effect of ${\alpha }_{{s}_{i}^{j}}$ and ${\alpha }_{{d}_{i}^{j}}$ for all alleles at locus $j$ from breed $b$, i.e. ${\epsilon }_{j}^{b}=E\left({\alpha }_{{s}_{i}^{j}}|{r}_{{s}_{i}^{j}}^{b}=1\right)=E\left({\alpha }_{{d}_{i}^{j}}|{r}_{{d}_{i}^{j}}^{b}=1\right)$. Furthermore, we define the within-breed additive effects of alleles as ${\alpha }_{{s}_{i}^{j}}^{b}={\alpha }_{{s}_{i}^{j}}-{\epsilon }_{j}^{b}$ when ${r}_{{s}_{i}^{j}}^{b}=1$ and ${\alpha }_{{d}_{i}^{j}}^{b}={\alpha }_{{d}_{i}^{j}}-{\epsilon }_{j}^{b}$, when ${r}_{{d}_{i}^{j}}^{b}=1$. The within-breed additive genetic effect of breed $b$ for individual $i$ is obtained by summing over all $m$ loci: ${a}_{i}^{b}={\sum }_{j=1}^{m}\left({r}_{{s}_{i}^{j}}^{b}{\alpha }_{{s}_{i}^{j}}^{b}+{r}_{{d}_{i}^{j}}^{b}{\alpha }_{{d}_{i}^{j}}^{b}\right)$. Similarly, the total contribution of the effects of local ancestry of alleles from breed $b$ for individual $i$ is obtained by summing over all $m$ loci: ${u}_{i}^{b}={\sum }_{j=1}^{m}{\epsilon }_{j}^{b}({r}_{{s}_{i}^{j}}^{b}+{r}_{{d}_{i}^{j}}^{b})$. The additive genetic value of individual $i$ can then be obtained by summing over all breeds:

2 $${g}_{i}=\sum_{b}{a}_{i}^{b}+\sum_{b}{u}_{i}^{b}.$$

The expectation of the total contribution of effects of local ancestry from breed $b$ to individual $i$ is $E\left({u}_{i}^{b}\right)= E\left[{\sum }_{j=1}^{m}{\epsilon }_{j}^{b}\left({r}_{{s}_{i}^{j}}^{b}+{r}_{{d}_{i}^{j}}^{b}\right)\right]={\sum }_{j=1}^{m}{\epsilon }_{j}^{b}\times E\left({r}_{{s}_{i}^{j}}^{b}+{r}_{{d}_{i}^{j}}^{b}\right)={\delta }^{b}{f}_{i}^{b},$

where ${\delta }^{b}={\sum }_{j=1}^{m}2{\epsilon }_{j}^{b}$ is the difference between the mean genetic level of breed $b$ and the general mean, and ${f}_{i}^{b}=\left({f}_{{s}_{i}}^{b}+{f}_{{d}_{i}}^{b}\right)/2=E\left({r}_{{s}_{i}^{j}}^{b}+{r}_{{d}_{i}^{j}}^{b}\right)/2$ is the expected proportion of genes in individual $i$ that originate from breed $b$. We can now modify Eq. (2) to ${g}_{i}=\sum_{b}{a}_{i}^{b}+\sum_{b}({\delta }^{b}\times {f}_{i}^{b})+\sum_{b}{\sum }_{j=1}^{m}{\epsilon }_{j}^{b}{\ddot{z}}_{{i}^{j}}^{b}$, where ${\ddot{z}}_{{i}^{j}}^{b}={z}_{{s}_{i}^{j}}^{b}+{z}_{{d}_{i}^{j}}^{b}$, ${z}_{{s}_{i}^{j}}^{b}={r}_{{s}_{i}^{j}}^{b}-{f}_{{s}_{i}}^{b}$, and ${z}_{{d}_{i}^{j}}^{b}={r}_{{d}_{i}^{j}}^{b}-{f}_{{d}_{i}}^{b}$ are deviations of the contribution of breed $b$ at locus $j$ from ${f}_{{s}_{i}}^{b}$ and ${f}_{{d}_{i}}^{b}$, the proportions of genes in ${s}_{i}$ and ${d}_{i}$, respectively, that originate from breed $b$.

Vector $\mathbf{g}$, which contains the additive genetic values for crossbred individuals involving ${n}_{b}$ breeds, can then be decomposed as:

3 $$\mathbf{g}=\sum_{b}{\mathbf{a}}^{b}+\sum_{b}({\delta }^{b}{\mathbf{f}}^{b})+\sum_{b}{\mathbf{k}}^{b},$$

where vector ${\mathbf{a}}^{b}$ contains the partial additive genetic effects related to breed $b$ as defined in García-Cortés and Toro [6] and Christensen et al. [5], with value ${a}_{i}^{b}={\sum }_{j=1}^{m}({r}_{{s}_{i}^{j}}^{b}{\alpha }_{{s}_{i}^{j}}^{b}+{r}_{{d}_{i}^{j}}^{b}{\alpha }_{{d}_{i}^{j}}^{b})$ for individual $i$, vector ${\mathbf{f}}^{b}$ contains ${f}_{i}^{b}$ for all individuals, and vector ${\mathbf{k}}^{b}$ contains ${k}_{i}^{b}={\sum }_{j=1}^{m}{\epsilon }_{j}^{b}{\ddot{z}}_{{i}^{j}}^{b}$. The effects of breed proportions, ${\delta }^{b}$ for each breed $b$, are fixed effects in the model and $\sum_{b}{\mathbf{a}}^{b}$ are random animal effects that are independent from $\sum_{b}{\mathbf{k}}^{b}$ [6, 9]. Therefore, the variance–covariance matrix of the vector of additive genetic values $\mathbf{g}$ for the crossbreds is therefore $Var\left(\mathbf{g}\right)=Var\left(\sum_{b}{\mathbf{a}}^{b}\right)+Var\left(\sum_{b}{\mathbf{k}}^{b}\right)$.

García-Cortés and Toro [6] modelled the term $\sum_{b}{\mathbf{k}}^{b}$ in Eq. (3) with BS terms, i.e. between each pair of breeds $b$ and ${b}^{{\prime}}$, instead of summing across breeds,

4 $$\mathbf{g}=\sum_{b}{\mathbf{a}}^{b}+\sum_{b}({\delta }^{b}{\mathbf{f}}^{b})+\sum_{b}\sum_{{b}^{{\prime}}>b}{\mathbf{w}}^{b,{b}^{{\prime}}},$$

where vector ${\mathbf{w}}^{b,{b}^{{\prime}}}$ contains the BS effects. In this study, we specify the (co)variance of the $\sum_{b}{\mathbf{k}}^{b}$ term in Eq. (3), i.e. $Var\left(\sum_{b}{\mathbf{k}}^{b}\right)$. Considering $Var\left(\sum_{b}\sum_{{b}^{{\prime}}>b}{\mathbf{w}}^{b,{b}^{{\prime}}}\right)=Var\left(\sum_{b}{\mathbf{k}}^{b}\right)$, we develop two alternative models for genetic evaluation of crossbreds, i.e. a model based on LBP terms for each breed and a model based on BS terms (Eq. 4).

### Breed-specific partial relationships

Here, we review how $\sum_{b}Var\left({\mathbf{a}}^{b}\right)$ is inferred from pedigree and genomic information in models where the breeding value of crossbreds is split into breed-specific parts. Pedigree-based breed-specific partial additive genetic (co)variance matrices were derived in García-Cortés and Toro [6] and breed-specific partial genomic relationship matrices for terminal three-way crossbreeding systems were derived in Christensen et al. [11] and Sevillano et al. [16].

Pedigree-based partial relationship matrices are constructed recursively, as described by García-Cortés and Toro [6]. For breed $b$, the self-relationship for individual $i$ is:

$${\mathbf{A}}_{i,i}^{b}={f}_{i}^{b}+\frac{1}{2}{\mathbf{A}}_{s,d}^{b},$$

and the partial relationship between individuals $i$ and ${i}^{{\prime}}$ is:

$${\mathbf{A}}_{i,{i}^{{\prime}}}^{b}=\frac{1}{2}\left({\mathbf{A}}_{s,{i}^{{\prime}}}^{b,}+{\mathbf{A}}_{d{,i}^{{\prime}}}^{b}\right),$$

where ${f}_{i}^{b}$ is the proportion of breed $b$, ${\mathbf{A}}_{s,d}^{b}$ is the partial relationship between the parents of $i$, and ${\mathbf{A}}_{s,{i}^{{\prime}}}^{b,}$ and ${\mathbf{A}}_{d,{i}^{{\prime}}}^{b,}$ are the partial relationships of individual ${i}^{{\prime}}$ with the sire and dam of $i$, respectively. Having the pedigree-based partial relationship matrices, the partial genetic effects in Eq. (3) are assumed to be independent and ${\mathbf{a}}^{b}\sim N\left(\mathbf{0},{\mathbf{A}}^{b}{\sigma }_{a\left(b\right)}^{2}\right)$, where ${\sigma }_{a(b)}^{2}$ is the additive genetic variance for breed $b$.

For crossbreds with genome-wide SNP information, we consider a genotype matrix $\mathbf{M}$ of size $n\times m$, where $n$ is the number of individuals and $m$ is the number of SNPs, containing genotype information coded as 0 and 2 for loci that are homozygous for the alternative and reference allele, respectively, and 1 for heterozygous loci. To connect the two alleles to the correct breed origin, we need to have the genotypes phased and split into contributions from maternal and paternal gametes. Therefore, $\mathbf{M}$ is split into paternal and maternal allele matrices, $\mathbf{M}={\mathbf{M}}_{s}+{\mathbf{M}}_{d}$, with the ${\mathbf{M}}_{s}$ and ${\mathbf{M}}_{d}$ matrices containing values equal to 1 for the reference allele and 0 for the alternative allele. Assigned BOA can give information on the origin of alleles throughout the genome and, thus, provides direct estimates of ${r}_{{s}_{i}^{j}}^{b}$ and ${r}_{{d}_{i}^{j}}^{b}$. From the assigned BOA, we can construct matrices ${\mathbf{T}}_{s}^{b}$ and ${\mathbf{T}}_{d}^{b}$ of size $n\times m$ that contain ${r}_{{s}_{i}^{j}}^{b}$ and ${r}_{{d}_{i}^{j}}^{b}$, respectively. By connecting the information on BOA and SNP genotypes, breed-specific genotype matrices can be formed: ${\mathbf{M}}^{b}={\mathbf{M}}_{s}\circ {\mathbf{T}}_{s}^{b}+{\mathbf{M}}_{d}\circ {\mathbf{T}}_{d}^{b}$, where $\circ$ is element-wise multiplication. The genomic partial relationship matrix for breed $b$ then is:

5 $${\mathbf{G}}^{b}=\frac{({\mathbf{M}}^{b}-\mathbf{1}{\left[{\mathbf{p}}^{b}\right]}^{T}\circ {\mathbf{T}}^{b}){({\mathbf{M}}^{b}-\mathbf{1}{\left[{\mathbf{p}}^{b}\right]}^{T}\circ {\mathbf{T}}^{b})}^{T}}{{c}^{b}},$$

where vector ${\mathbf{p}}^{b}$ contains the allele frequencies of the alternative allele in breed $b$, ${\mathbf{T}}^{b}={\mathbf{T}}_{s}^{b}+{\mathbf{T}}_{d}^{b}$, and ${c}^{b}$ is a scaling parameter that can be chosen such that the partial genomic matrix is compatible with the pedigree partial relationship matrix [5]. Based on the genomic partial relationship matrices, the partial genetic effects in Eq. (3) are assumed to be distributed ${\mathbf{a}}^{b}\sim N\left(\mathbf{0},{\mathbf{G}}^{b}{\sigma }_{a(b)}^{2}\right)$.

### Breed segregation

For the pedigree-based model, García-Cortés and Toro [6] derived the distribution for the ${\mathbf{w}}^{b,{b}^{{\prime}}}$ term in Eq. (4) to be ${\mathbf{w}}^{b,{b}^{{\prime}}}\sim N\left(\mathbf{0},{\mathbf{A}}_{(w)}^{b,{b}^{{\prime}}}{\sigma }_{w(b,{b}^{{\prime}})}^{2}\right)$, where (w) indicates BS, ${\mathbf{A}}_{(w)}^{b,{b}^{{\prime}}}$ is the pedigree-based partial relationship matrix for BS between breeds $b$ and $b^{\prime}$, and ${\sigma }_{w(b,{b}^{{\prime}})}^{2}$ is the BS variance between the two breeds. Matrix ${\mathbf{A}}_{(w)}^{b,{b}^{{\prime}}}$ is constructed based on:

$${\mathbf{A}}_{(w)i,i}^{b,{b}^{{\prime}}}=2{f}_{{s}_{i}}^{b}{f}_{{s}_{i}}^{{b}^{{\prime}}}+2{f}_{{d}_{i}}^{b}{f}_{{d}_{i}}^{{b}^{{\prime}}}+\frac{1}{2}{\mathbf{A}}_{(w)s,d}^{b,{b}^{{\prime}}},$$

and

6 $${\mathbf{A}}_{(w)i,{i}^{{\prime}}}^{b,{b}^{{\prime}}}=\frac{1}{2}\left({\mathbf{A}}_{\left(w\right)s,{i}^{{\prime}}}^{b,{b}^{{\prime}}}+{\mathbf{A}}_{\left(w\right)d{,i}^{{\prime}}}^{b,{b}^{{\prime}}}\right),$$

where ${f}_{{s}_{i}}^{{b}^{{\prime}}}$ and ${f}_{{d}_{i}}^{{b}^{{\prime}}}$ are the proportions of breed ${b}^{{\prime}}$ for the sire $s$ and dam $d$ of $i$*,* respectively [6].

We now consider the BS variance structure based on the BOA information in ${\mathbf{T}}^{b}$. We define ${\kappa }_{{s}_{i}^{j}}^{b}={\epsilon }_{j}^{b}{z}_{{s}_{i}^{j}}^{b}$, ${\kappa }_{{d}_{i}^{j}}^{b}={\epsilon }_{j}^{b}{z}_{{d}_{i}^{j}}^{b}$, ${\kappa }_{{s}_{i}^{j}}^{sum}=\sum_{b}{\kappa }_{{s}_{i}^{j}}^{b}$, ${\kappa }_{{d}_{i}^{j}}^{sum}=\sum_{b}{\kappa }_{{d}_{i}^{j}}^{b}$, and ${\ddot{\kappa }}_{{i}^{j}}^{b}={\epsilon }_{j}^{b}{\ddot{z}}_{{i}^{j}}^{b}$. To derive the formulas for genomic models, we assume that the markers are the QTL in Eq. (1), rather than markers that are in linkage disequilibrium with the QTL. We further assume the local breed origin to be known for the markers and we assume the effect of local ancestry ${\epsilon }_{j}^{b}$ to be a random variable (as in Christensen et al. [11]), with mean zero. For a single locus, the total BS variance is: $Var\left({\ddot{\kappa }}_{{i}^{j}}^{sum}\right)=E\left[{\left(\sum_{b}{\epsilon }_{j}^{b}{\ddot{z}}_{{i}^{j}}^{b}\right)}^{2}\right]$.

After some algebra, we get (see derivation in Appendix 1):

7 $$Var\left({\ddot{\kappa }}_{{i}^{j}}^{sum}\right)=\sum_{b}\sum_{{b}^{{\prime}}>b}-{\ddot{z}}_{{i}^{j}}^{b}{\ddot{z}}_{{i}^{j}}^{{b}^{{\prime}}}E\left[{\left({\epsilon }_{j}^{b}-{\epsilon }_{j}^{{b}^{{\prime}}}\right)}^{2}\right].$$

Similarly, the breed-segregation covariance between individuals $i$ and $i^{\prime}$ for one locus is (see derivation in Appendix 1):

8 $$Cov\left({\ddot{\kappa }}_{{i}^{j}}^{sum},{\ddot{\kappa }}_{{{i}^{{\prime}}}^{j}}^{sum}\right)=\sum_{b}\sum_{{b}^{{\prime}}>b}\left(-{\ddot{z}}_{{i}^{j}}^{b}{\ddot{z}}_{{{i}^{{\prime}}}^{j}}^{{b}^{{\prime}}}-{\ddot{z}}_{{i}^{j}}^{{b}^{{\prime}}}{\ddot{z}}_{{{i}^{{\prime}}}^{j}}^{b}\right)\frac{1}{2}E\left[{\left({\epsilon }_{j}^{b}-{\epsilon }_{j}^{{b}^{{\prime}}}\right)}^{2}\right].$$

Assuming ${\epsilon }_{j}^{b}$ and ${\epsilon }_{{j}^{{\prime}}}^{{b}^{{\prime}}}$ are independent when $j\ne {j}^{{\prime}}$, both for $b={b}^{{\prime}}$ and for $b\ne {b}^{{\prime}}$, and that each locus contributes equally to the total BS variance for each pair of breeds, we extend the result from the single locus in Eqs. (7) and (8) to the whole genome. We then have $Var\left({k}_{i}^{sum}\right)=\sum_{b}\sum_{{b}^{{\prime}}>b}-\frac{1}{m}{{\mathbf{z}}_{i}^{b}}^{T}{\mathbf{z}}_{i}^{{b}^{{\prime}}}\sum_{j=1}^{m}E\left[{\left({\epsilon }_{j}^{b}-{\epsilon }_{j}^{{b}^{{\prime}}}\right)}^{2}\right]$ and $Cov\left({k}_{i}^{sum},{k}_{{i}^{{\prime}}}^{sum}\right)=\sum_{b}\sum_{{b}^{{\prime}}>b}-\frac{1}{2m}\left({{\mathbf{z}}_{i}^{b}}^{T}{\mathbf{z}}_{{i}^{{\prime}}}^{{b}^{{\prime}}}+{{\mathbf{z}}_{i}^{{b}^{{\prime}}}}^{T}{\mathbf{z}}_{{i}^{{\prime}}}^{b}\right)\sum_{j=1}^{m}E\left[{\left({\epsilon }_{j}^{b}-{\epsilon }_{j}^{{b}^{{\prime}}}\right)}^{2}\right]$, where ${\mathbf{z}}_{i}^{b}$ is a vector of ${\ddot{z}}_{{i}^{j}}^{b}$ for all loci for individual $i$, and similarly for breed ${b}^{{\prime}}$ and individual ${i}^{{\prime}}$. Realizing that ${{\mathbf{z}}_{i}^{b}}^{T}{\mathbf{z}}_{i}^{{b}^{{\prime}}}=\frac{1}{2}({{\mathbf{z}}_{i}^{b}}^{T}{\mathbf{z}}_{i}^{{b}^{{\prime}}}+{{\mathbf{z}}_{i}^{{b}^{{\prime}}}}^{T}{\mathbf{z}}_{i}^{b})$, we write the BS (co)variance as $Var\left(\sum_{b}\sum_{{b}^{{\prime}}>b}{\mathbf{w}}^{b,{b}^{{\prime}}}\right)=\sum_{b}\sum_{{b}^{\boldsymbol{^{\prime}}}>b}{\mathbf{W}}^{b,{b}^{{\prime}}}{\sigma }_{w(b,{b}^{{\prime}})}^{2}$, where

9 $${\mathbf{W}}^{b,{b}^{{\prime}}}=-\frac{{\mathbf{Z}}^{b}{{\mathbf{Z}}^{{b}^{{\prime}}}}^{T}+{\mathbf{Z}}^{{b}^{{\prime}}}{{\mathbf{Z}}^{b}}^{T}}{2m}$$

is a BS similarity matrix for BS between breeds $b$ and $b^{\prime}$, with ${\mathbf{Z}}^{b}={\mathbf{T}}^{b}-2{\mathbf{f}}^{b}{\mathbf{1}}^{T}$, and $\mathbf{1}$ is a vector of 1s, and

10 $${\sigma }_{w(b,{b}^{{\prime}})}^{2}=\frac{1}{2}\sum_{j=1}^{m}E\left[{\left({\epsilon }_{j}^{b}-{\epsilon }_{j}^{{b}^{{\prime}}}\right)}^{2}\right]$$

is the variance for BS between breeds $b$ and $b^{\prime}$. Here, the definition of the breed-segregation variance follows, e.g., Lo et al. [4], and is defined as the extra genetic variance in the F2 population compared to the F1.

However, matrix ${\mathbf{W}}^{b,{b}^{{\prime}}}$ does not have a quadratic form and is, thus, not necessarily positive semi-definite. Thus, the BOA-derived BS similarity matrix is not a proper relationship matrix. In contrast, the pedigree-based partial BS relationship matrix given by García-Cortés and Toro [6] is always positive semi-definite.

### Genomic local breed proportion (co)variance

Eiríksson et al. [14] fitted three independent LBP effects in a genomic model for a population of crossbreds from three breeds. The effects were either fitted with random regression on LBP (similar to SNP-best linear unbiased prediction (BLUP) for markers) or using LBP similarity matrices. In Appendix 2, we show that the variance for LBP effects from fitting three independent LBP terms is not guaranteed to be non-negative and, therefore, not a valid variance term. Here, we present an alternative LBP model, where one of the breeds is set as a reference breed and is left out of the model, but the other LBP effects are assumed to be correlated. We start by presenting a genomic model based on BOA, followed by the pedigree-based model. As before, we assume that the marker loci are the QTL, the origin of alleles is known, and the ${\epsilon }_{j}^{b}$ terms are random unknown variables with mean zero.

The variance for LBP effects over all breeds $b$ for locus $j$ is $Var\left({\ddot{\kappa }}_{{i}^{j}}^{sum}\right)=E\left[{\left(\sum_{b}{{\ddot{z}}_{{i}^{j}}^{b}\epsilon }_{j}^{b}\right)}^{2}\right]$. We set ${b}^{*}$ as the reference breed. For breeds $b\ne {b}^{*}$, and ${b}^{{\prime}}\ne {b}^{*}$, we substitute ${\epsilon }_{j}^{b}={\epsilon }_{j}^{{b}^{*}}+{\epsilon }_{j}^{b}-{\epsilon }_{j}^{{b}^{*}}$. After algebra detailed in Appendix 3, we have:

11 $$Var\left({\ddot{\kappa }}_{{i}^{j}}^{sum}\right)=\sum_{b\ne {b}^{*}}\sum_{{b}^{{\prime}}\ne {b}^{*}}{\ddot{z}}_{{i}^{j}}^{b}{\ddot{z}}_{{i}^{j}}^{{b}^{{\prime}}}E\left[\left({\epsilon }_{j}^{b}-{\epsilon }_{j}^{{b}^{*}}\right)\left({\epsilon }_{j}^{{b}^{{\prime}}}-{\epsilon }_{j}^{{b}^{*}}\right)\right].$$

Now, we have ${n}_{b}-1$ LBP variance terms and their covariances between breeds [the $b\ne {b}^{{\prime}}$ case in Eq. (11)]. The covariance between the LBP effects of individuals $i$ and ${i}^{{\prime}}$ comes from the deviation in shared ancestry of loci from the expectation based on breed proportions, which is derived in the same manner as for the LBP variance above (see Appendix 3). Therefore,

12 $$Cov\left({\ddot{\kappa }}_{{i}^{j}}^{sum},{\ddot{\kappa }}_{{{i}^{{\prime}}}^{j}}^{sum}\right)=\sum_{b\ne {b}^{*}}\sum_{{b}^{{\prime}}\ne {b}^{*}}{\ddot{z}}_{{i}^{j}}^{b}{\ddot{z}}_{{{i}^{{\prime}}}^{j}}^{{b}^{{\prime}}}E\left[\left({\epsilon }_{j}^{b}-{\epsilon }_{j}^{{b}^{*}}\right)\left({\epsilon }_{j}^{{b}^{{\prime}}}-{\epsilon }_{j}^{{b}^{*}}\right)\right].$$

Assuming that ${\epsilon }_{j}^{b}$ and ${\epsilon }_{{j}^{{\prime}}}^{{b}^{{\prime}}}$ are independent for $j\ne {j}^{{\prime}}$, both when $b={b}^{{\prime}}$ and when $b\ne {b}^{{\prime}}$, the total variance of LBP effects related to individual $i$ is obtained by summing the contributions of each locus from Eq. (11):

13 $$Var\left({k}_{i}^{sum}\right)=\sum_{b\ne {b}^{*}}\sum_{{b}^{{\prime}}\ne {b}^{*}}\sum_{j=1}^{m}{\ddot{z}}_{{i}^{j}}^{b}{\ddot{z}}_{{i}^{j}}^{{b}^{{\prime}}}E\left[\left({\epsilon }_{j}^{b}-{\epsilon }_{j}^{{b}^{*}}\right)\left({\epsilon }_{j}^{{b}^{{\prime}}}-{\epsilon }_{j}^{{b}^{*}}\right)\right].$$

Similar to the SNP-BLUP and genomic BLUP models, we assume that the contribution of the (co)variance of LBP effects is the same for each locus for each breed and we denote this by ${\sigma }_{\theta (b{b}^{{\prime}})}$. That is, for all loci $j$ and ${j}^{{\prime}}$,

14 $${\sigma }_{\theta \left(b{b}^{{\prime}}\right)}=E\left[\left({\epsilon }_{j}^{b}-{\epsilon }_{j}^{{b}^{*}}\right)\left({\epsilon }_{j}^{{b}^{{\prime}}}-{\epsilon }_{j}^{{b}^{*}}\right)\right]=E\left[\left({\epsilon }_{{j}^{{\prime}}}^{b}-{\epsilon }_{{j}^{{\prime}}}^{{b}^{*}}\right)\left({\epsilon }_{{j}^{{\prime}}}^{{b}^{{\prime}}}-{\epsilon }_{{j}^{{\prime}}}^{{b}^{*}}\right)\right].$$

Therefore, we can write Eq. (13) as:

15 $$Var\left({k}_{i}^{sum}\right)=\sum_{b\ne {b}^{*}}\sum_{{b}^{{\prime}}\ne {b}^{*}}{{\mathbf{z}}_{i}^{b}}^{T}{\mathbf{z}}_{i}^{{b}^{{\prime}}}{\sigma }_{\theta (b{b}^{{\prime}})}.$$

Similarly, the covariance of LBP effects between individuals $i$ and ${i}^{{\prime}}$ from summing over loci based on Eqs. (12) and (14) is:

16 $$Cov\left({k}_{i}^{sum},{k}_{{i}^{{\prime}}}^{sum}\right)=\sum_{b\ne {b}^{*}}\sum_{{b}^{{\prime}}\ne {b}^{*}}{{\mathbf{z}}_{i}^{b}}^{T}{\mathbf{z}}_{{i}^{{\prime}}}^{{b}^{{\prime}}}{\sigma }_{\theta (b{b}^{{\prime}})}.$$

Combining Eqs. (15) and (16), and extending to multiple individuals, we write the total (co)variance of LBP effects as $Var\left(\sum_{b}{\mathbf{k}}^{b}\right)=\sum_{b\ne {b}^{*}}\sum_{{b}^{\boldsymbol{^{\prime}}}\ne {b}^{*}}{\mathbf{Q}}^{b{b}^{{\prime}}}{\sigma }_{k(b{b}^{{\prime}})}$, where

17 $${\mathbf{Q}}^{b{b}^{{\prime}}}=\frac{{\mathbf{Z}}^{b}{{\mathbf{Z}}^{{b}^{{\prime}}}}^{T}}{m}$$

is a partial relationship matrix for LBP effects within breed when $b = b^{\prime}$ and a matrix describing relationships of LBP effects between breeds $b$ and $b^{\prime}$ when $b \ne b^{\prime}$. Furthermore,

18 $${\sigma }_{k(b{b}^{{\prime}})}=m{\sigma }_{\theta (b{b}^{{\prime}})}=\sum_{j=1}^{m}E\left[\left({\epsilon }_{j}^{b}-{\epsilon }_{j}^{{b}^{*}}\right)\left({\epsilon }_{j}^{{b}^{{\prime}}}-{\epsilon }_{j}^{{b}^{*}}\right)\right]$$

is the variance of LBP effects for breed $b$ when $b = b^{\prime}$ and the covariance between LBP effects for breeds $b$ and $b^{\prime}$ when $b \ne b^{\prime}$.

When $b={b}^{{\prime}}$, both the partial relationship matrix (${\mathbf{Q}}^{bb}$) and the variance component (${\sigma }_{k(bb)}$) have a quadratic form and are therefore a valid relationship matrix and variance component, respectively. Defined in this way, the diagonal elements of ${\mathbf{Q}}^{bb}$ depend on deviations from the global breed proportion of individuals from their assignment to breed $b$ of individual SNPs. The off-diagonal element between two individuals measures the degree to which these individuals share local deviations from the global breed proportions for breed $b$. For $b\ne {b}^{{\prime}}$, the ${\mathbf{Q}}^{b{b}^{{\prime}}} {\sigma }_{k(b{b}^{{\prime}})}$ and ${\mathbf{Q}}^{{b}^{{\prime}}b} {\sigma }_{k(b{b}^{{\prime}})}$ terms contain covariances between the LBP effects for breeds $b$ and ${b}^{{\prime}}$ for all pairs of individuals (where the diagonal elements are covariances between breeds within individuals). Note that ${\mathbf{Q}}^{b{b}^{{\prime}}}={{\mathbf{Q}}^{{b}^{{\prime}}b}}^{T}$, while ${\sigma }_{k(b{b}^{{\prime}})}={\sigma }_{k({b}^{{\prime}}b)}$.

Using the variance structure described above, we write the total additive genetic effect in Eq. (3) based on ${n}_{b}-1$ modified LBP effects:

19 $$\mathbf{g}=\sum_{b}\left({\mathbf{a}}^{b}+{\delta }^{b}{\mathbf{f}}^{b}\right)+\sum_{b\ne {b}^{*}}{\widetilde{\mathbf{k}}}^{b},$$

where vector ${\widetilde{\mathbf{k}}}^{b}$ contains the modified LBP effects of breed $b$, and we assume that:

20 $$\left[\begin{array}{c}{\widetilde{\mathbf{k}}}^{1}\\ \vdots \\ {\widetilde{\mathbf{k}}}^{{n}_{b}-1}\end{array}\right]\sim N\left(\mathbf{0},\left[\begin{array}{ccc}{\mathbf{Q}}^{11}{\sigma }_{k(11)}& \cdots & {\mathbf{Q}}^{{n}_{b}-\mathrm{1,1}}{\sigma }_{k(1, {n}_{b}-1)}\\ \vdots & \ddots & \vdots \\ {\mathbf{Q}}^{1{n}_{b}-1}{\sigma }_{k(1, {n}_{b}-1)}& \cdots & {\mathbf{Q}}^{{n}_{b}-1,{n}_{b}-1}{\sigma }_{k({n}_{b}-1,{n}_{b}-1)}\end{array}\right]\right).$$

In Eq. (20), the combined (co)variance structure for the LBP effects has a quadratic form and is therefore a valid (co)variance structure. Furthermore, the combined relationship matrix for LBP effects, $$\mathbf{Q}=\left[\begin{array}{ccc}{\mathbf{Q}}^{11}& \cdots & {\mathbf{Q}}^{{n}_{b}-\mathrm{1,1}}\\ \vdots & \ddots & \vdots \\ {\mathbf{Q}}^{1{n}_{b}-1}& \cdots & {\mathbf{Q}}^{{n}_{b}-1,{n}_{b}-1}\end{array}\right],$$ also has a quadratic form, similar to a genomic relationship matrix [1]. However, the variance and covariance components cannot be factored directly out of the combined LBP (co)variance matrices. The variance structure in Eq. (20) can alternatively be expressed as:

21 $$Var\left[\begin{array}{c}\begin{array}{c}{\widetilde{\mathbf{k}}}^{1}\\ \vdots \\ {\widetilde{\mathbf{k}}}_{\star }^{{n}_{b}-1, 1}\end{array}\\ \vdots \\ \begin{array}{c}{\widetilde{\mathbf{k}}}_{\star }^{1,{n}_{b}-1}\\ \vdots \\ {\widetilde{\mathbf{k}}}^{{n}_{b}-1}\end{array}\end{array}\right]={\varvec{\Sigma}}\otimes \mathbf{Q},$$

where ${\mathbf{k}}_{\star }^{b,{b}^{\boldsymbol{^{\prime}}}}$, for $b$ and ${b}^{{\prime}}$ in (1,…, ${n}_{b}-1$), are vectors with artificial variables, i.e. variables that are not meaningful in themselves but included only to make the expression as a Kronecker product possible, matrix $${\varvec{\Sigma}}=\left[\begin{array}{ccc}{\sigma }_{k(11)}& \cdots & {\sigma }_{k(1, {n}_{b}-1)}\\ \vdots & \ddots & \vdots \\ {\sigma }_{k(1, {n}_{b}-1)}& \cdots & {\sigma }_{k({n}_{b}-1,{n}_{b}-1)}\end{array}\right]$$ is positive definite, and $\otimes$ is Kronecker product. Based on this formulation, the variance components for LBP effects can be estimated using standard software.

Similar to usual genomic BLUP, an equivalent SNP-BLUP model can be formed from the model in Eq. (19):

$$\mathbf{g}=\sum_{b}\left({\mathbf{M}}^{b}{{\varvec{\upalpha}}}^{b}+{\delta }^{b}{\mathbf{f}}^{b}\right)+\sum_{b\ne {b}^{*}}{\mathbf{Z}}^{b}{{\varvec{\uptheta}}}^{b},$$

where vector ${{\varvec{\upalpha}}}^{b}$ contains marker effects in breed $b$ and vector ${{\varvec{\uptheta}}}^{b}$ contains the modified effects of LBP from breed $b$ for each marker. It is assumed that ${{\varvec{\upalpha}}}^{b}\sim N(\mathbf{0},\mathbf{I}\frac{1}{{c}^{b}}{\sigma }_{a\left(b\right)}^{2})$ (${c}^{b}$ is the scaling factor used in building ${\mathbf{G}}^{b}$) and $$\left[\begin{array}{c}{{\varvec{\uptheta}}}_{x}^{1}\\ \vdots \\ {{\varvec{\uptheta}}}_{x}^{{n}_{b}-1}\end{array}\right]\sim N\left(\mathbf{0},\left[\begin{array}{ccc}\mathbf{I}{\sigma }_{\theta (11)}& \cdots & \mathbf{I}{\sigma }_{\theta (1{n}_{b}-1)}\\ \vdots & \ddots & \vdots \\ \mathbf{I}{\sigma }_{\theta (1{n}_{b}-1)}& \cdots & \mathbf{I}{\sigma }_{\theta ({n}_{b}-1,{n}_{b}-1)}\end{array}\right]\right)$$.

### Pedigree-based local breed proportion (co)variance

Here, we present how the (co)variance structure for LBP effects with one breed as a reference breed can be constructed using pedigree information. First, consider the contribution to the variance of the LBP effect from the paternal allele at a single locus. The expectation that the paternal allele at locus $j$ is from breed $b$ equals the sire’s breed proportion, i.e. $E\left({r}_{{s}_{i}^{j}}^{b}\right)={f}_{{s}_{i}}^{b}$. Therefore, the expectation of ${\kappa }_{{s}_{i}^{j}}^{sum}$ is $E\left({\kappa }_{{s}_{i}^{j}}^{sum}\right)=\sum_{b}{\epsilon }_{j}^{b}{f}_{{s}_{i}}^{b}$. For the variance related to the LBP effect of the paternal allele, ${r}_{{s}_{i}^{j}}^{b}{r}_{{s}_{i}^{j}}^{{b}^{{\prime}}}=0$ for all $b\ne {b}^{{\prime}}$, because the paternal allele at locus $j$ originates only from one of the breeds. Furthermore, ${({r}_{{s}_{i}^{j}}^{b})}^{2}={r}_{{s}_{i}^{j}}^{b}$ for all $b$, because ${r}_{{s}_{i}^{j}}^{b}$ is either 0 or 1. Therefore, $E\left[Var\left({\kappa }_{{s}_{i}^{j}}^{sum}\right)\right]=\sum_{b}{f}_{{s}_{i}}^{b}{{\epsilon }_{j}^{b}}^{2}-{\left(\sum_{b}{f}_{{s}_{i}}^{b}{\epsilon }_{j}^{b}\right)}^{2}$. By setting one breed as the reference breed, ${b}^{*}$, and with algebra as detailed in Appendix 4, we have ${n}_{b}-1$ LBP terms for expected variance of the LBP effect of the allele (for $b={b}^{{\prime}}$),

22 $$E\left[Var\left({\kappa }_{{s}_{i}^{j}}^{b}\right)\right]=\sum_{b\ne {b}^{*}}{f}_{{s}_{i}}^{b}\left(1-{f}_{{s}_{i}}^{b}\right){\left({\epsilon }_{j}^{b}-{\epsilon }_{j}^{{b}^{*}}\right)}^{2}.$$

Furthermore, based on algebra described in Appendix 4, the covariance between the LBP effects for breeds $b$ and ${b}^{{\prime}}$ is:

23 $$E\left[Cov\left({\kappa }_{{s}_{i}^{j}}^{b},{\kappa }_{{s}_{i}^{j}}^{{b}^{{\prime}}}\right)\right]=\sum_{b\ne {b}^{*}}\sum_{{b}^{{\prime}}\ne \left\{b,{b}^{*}\right\}}\left[-{f}_{{s}_{i}}^{b}{f}_{{s}_{i}}^{{b}^{{\prime}}}\left({\epsilon }_{j}^{{b}^{{\prime}}}-{\epsilon }_{j}^{{b}^{*}}\right)\left({\epsilon }_{j}^{b}-{\epsilon }_{j}^{{b}^{*}}\right)\right].$$

Similarly, for the maternal allele,

24 $$E\left[Var\left({\kappa }_{{d}_{i}^{j}}^{b}\right)\right]=\sum_{b\ne {b}^{*}}{f}_{{d}_{i}}^{b}(1-{f}_{{d}_{i}}^{b}){({\epsilon }_{j}^{b}-{\epsilon }_{j}^{{b}^{*}})}^{2},$$

and

25 $$E\left[Cov\left({\kappa }_{{d}_{i}^{j}}^{b},{\kappa }_{{d}_{i}^{j}}^{{b}^{{\prime}}}\right)\right]=\sum_{b\ne {b}^{*}}\sum_{{b}^{{\prime}}\ne \left\{b,{b}^{*}\right\}}\left[-{f}_{{d}_{i}}^{b}{f}_{{d}_{i}}^{{b}^{{\prime}}}\left({\epsilon }_{j}^{{b}^{{\prime}}}-{\epsilon }_{j}^{{b}^{*}}\right)\left({\epsilon }_{j}^{b}-{\epsilon }_{j}^{{b}^{*}}\right)\right].$$

For the expectation of the variance of LBP effects related to both alleles, we need the covariance between the maternal and paternal LBP effects for locus $j$ in individual $i$. The probability that the paternal allele of individual $i$ comes from the paternal grandsire (or granddam) is $\frac{1}{2}$, and thus,

26 $$E\left({\kappa }_{{s}_{i}^{j}}^{sum}\right)=\frac{1}{2}\left({\kappa }_{{s}_{s}^{j}}^{sum}+{\kappa }_{{d}_{s}^{j}}^{sum}\right)=\frac{1}{2}{\ddot{\kappa }}_{{s}^{j}}^{sum}.$$

Similarly, $E({\kappa }_{{d}_{i}^{j}}^{sum})=\frac{1}{2}({\kappa }_{{s}_{d}^{j}}^{sum}+{\kappa }_{{d}_{d}^{j}}^{sum})=\frac{1}{2}{\ddot{\kappa }}_{{d}^{j}}^{sum}$. Therefore, the expectation of the covariance between the LBP effects of the maternal and paternal alleles is:

27 $$\text{E}\left[Cov\left({\kappa }_{{s}_{i}^{j}}^{sum},{\kappa }_{{d}_{i}^{j}}^{sum}\right)\right]=\frac{1}{4}Cov\left({\ddot{\kappa }}_{{s}^{j}}^{sum},{\ddot{\kappa }}_{{d}^{j}}^{sum}\right),$$

where $Cov({\ddot{\kappa }}_{{s}^{j}}^{sum},{\ddot{\kappa }}_{{d}^{j}}^{sum})$ is the covariance between LBP effects at locus $j$ of the two parents of $i$.

The expectation of the covariance of the LBP effects of the maternal and paternal alleles is thus $1/4$ of the covariance between LBP effects of the parents, which is a known result for pedigree and BS relationships [6].

Now, we consider the LBP covariance between individuals $i$ and ${i}^{{\prime}}$. First, we look at the covariance of paternal allele of individual $i$ at locus $j$ with ${\ddot{\kappa }}_{{{i}^{{\prime}}}^{j}}^{sum}$. Using the same argument as for Eq. (26), we have $E\left[Cov\left({\kappa }_{{s}_{i}^{j}}^{sum},{\ddot{\kappa }}_{{{i}^{{\prime}}}^{j}}^{sum}\right)\right]=\frac{1}{2}Cov\left({\ddot{\kappa }}_{{s}^{j}}^{sum},{\ddot{\kappa }}_{{{i}^{{\prime}}}^{j}}^{sum}\right)$. Similar to Eq. (11), we can write $Cov\left({\ddot{\kappa }}_{{s}^{j}}^{sum},{\ddot{\kappa }}_{{{i}^{{\prime}}}^{j}}^{sum}\right)=\sum_{b\ne {b}^{*}}\sum_{{b}^{{\prime}}\ne {b}^{*}}{\ddot{z}}_{{s}^{j}}^{b}{\ddot{z}}_{{{i}^{{\prime}}}^{j}}^{{b}^{{\prime}}}\left({\epsilon }_{j}^{b}-{\epsilon }_{j}^{{b}^{*}}\right)\left({\epsilon }_{j}^{{b}^{{\prime}}}-{\epsilon }_{j}^{{b}^{*}}\right)$, i.e. $Cov\left({\ddot{\kappa }}_{{s}^{j}}^{sum},{\ddot{\kappa }}_{{{i}^{{\prime}}}^{j}}^{sum}\right)=\sum_{b\ne {b}^{*}}\sum_{{b}^{{\prime}}\ne {b}^{*}}Cov\left({\ddot{\kappa }}_{{s}^{j}}^{b},{\ddot{\kappa }}_{{{i}^{{\prime}}}^{j}}^{{b}^{{\prime}}}\right)$. Therefore, $E\left[Cov\left({\kappa }_{{s}_{i}^{j}}^{sum},{\ddot{\kappa }}_{{{i}^{{\prime}}}^{j}}^{sum}\right)\right]$=$\sum_{b\ne {b}^{*}}\sum_{{b}^{{\prime}}\ne {b}^{*}}\frac{1}{2}\left[Cov\left({\ddot{\kappa }}_{{s}^{j}}^{b},{\ddot{\kappa }}_{{{i}^{{\prime}}}^{j}}^{{b}^{{\prime}}}\right)\right]$ and, similarly, $E\left[Cov\left({\kappa }_{{d}_{i}^{j}}^{sum},{\ddot{\kappa }}_{{{i}^{{\prime}}}^{j}}^{sum}\right)\right]= \sum_{b\ne {b}^{*}}\sum_{{b}^{{\prime}}\ne {b}^{*}}\frac{1}{2}\left[Cov\left({\ddot{\kappa }}_{{d}^{j}}^{b},{\ddot{\kappa }}_{{{i}^{{\prime}}}^{j}}^{{b}^{{\prime}}}\right)\right]$. Therefore,

$$\begin{aligned} E\left[ {Cov\left( {\ddot{\kappa }_{{i^{j} }}^{sum} ,\ddot{\kappa }_{{i^{\prime{j}}}}^{sum} } \right)} \right] & = E\left[ {Cov\left( {\kappa_{{s_{i}^{j} }}^{sum} ,\ddot{\kappa }_{{i^{\prime{j}}}}^{sum} } \right)} \right] + E\left[ {Cov\left( {\kappa_{{d_{i}^{j} }}^{sum} ,\ddot{\kappa }_{{i^{\prime{j}}}}^{sum} } \right)} \right] \\ & = \sum \limits_{{b \ne b^{*} }} \sum \limits_{{b^{\prime} \ne b^{*} }} \frac{1}{2}\left[ {Cov\left( {\ddot{\kappa }_{{s^{j} }}^{b} ,\ddot{\kappa }_{{i^{\prime{j}}}}^{{b^{\prime}}} } \right) + Cov\left( {\ddot{\kappa }_{{d^{j} }}^{b} ,\ddot{\kappa }_{{i^{\prime{j}}}}^{{b^{\prime}}} } \right)} \right]. \\ \end{aligned}$$

Summing over all loci and the paternal and maternal alleles in Eqs. (22) to (25) and Eq. (27) gives the expected variance of LBP effects for individual $i$:

28 $$\begin{aligned} E\left[ {Var\left( {k_{i}^{sum} } \right)} \right] & = \sum \limits_{{b \ne b^{*} }} \left( {\left[ {f_{{s_{i} }}^{b} \left( {1 - f_{{s_{i} }}^{b} } \right) + f_{{d_{i} }}^{b} \left( {1 - f_{{d_{i} }}^{b} } \right)} \right] \sum \limits_{j = 1}^{m} \left( {\epsilon_{j}^{b} - \epsilon_{j}^{{b^{*} }} } \right)^{2} + \frac{1}{2}Cov\left( {k_{{s_{i} }}^{b} ,k_{{d_{i} }}^{b} } \right)} \right) \\ & + \sum \limits_{{b \ne b^{*} }} \sum \limits_{{b^{\prime} \ne \left\{ {b,b^{*} } \right\}}} \left[ {\left( { - f_{{s_{i} }}^{b} f_{{s_{i} }}^{{b^{\prime}}} - f_{{s_{i} }}^{b} f_{{s_{i} }}^{{b^{\prime}}} } \right) \sum \limits_{j = 1}^{m} \left( {\epsilon_{j}^{{b^{\prime}}} - \epsilon_{j}^{{b^{*} }} } \right)\left( {\epsilon_{j}^{b} - \epsilon_{j}^{{b^{*} }} } \right)} \right] + \frac{1}{4}Cov\left( {k_{{s_{i} }}^{b} ,k_{{d_{i} }}^{{b^{\prime}}} } \right) + \frac{1}{4}Cov\left( {k_{{s_{i} }}^{{b^{\prime}}} ,k_{{d_{i} }}^{b} } \right) \\ \end{aligned}$$

and the expected covariance between individuals becomes:

29 $$E\left[ {Cov\left( {k_{i}^{{sum}} ,k_{{i^{\prime } }}^{{sum}} } \right)} \right] = \sum\nolimits_{{b \ne b^{*} }} {\sum\nolimits_{{b^{\prime } \ne b^{*} }} {\frac{1}{2}} } \left[ {Cov\left( {k_{{s_{i} }}^{{sum}} ,k_{{i^{\prime } }}^{{sum}} } \right) + Cov\left( {k_{{d_{i} }}^{{sum}} ,k_{{i^{\prime } }}^{{sum}} } \right)} \right].$$

Combining Eqs. (28) and (29), and extending to multiple individuals, we write the (co)variance of LBP effects as $Var\left(\sum_{b}{\mathbf{k}}^{b}\right)=\sum_{b\ne {b}^{*}}\sum_{{b}^{\boldsymbol{^{\prime}}}\ne {b}^{*}}{\mathbf{A}}_{(k)}^{b{b}^{{\prime}}}{\sigma }_{k(b{b}^{{\prime}})}$, where ${\mathbf{A}}_{(k)}^{b,{b}^{{\prime}}}$ is a pedigree-based partial LBP relationship matrix when $b = b^{\prime}$ and a matrix that describes the relationship between the LBP effects of breeds $b$ and $b^{\prime}$ when $b \ne b^{\prime}$. Lo et al. [4] showed that pedigree relationship matrices that include BS can be constructed using the usual recursive rules, which, in turn, García-Cortés and Toro [6] used to construct partial BS relationship matrices. Similarly, the partial LBP relationship matrices ${\mathbf{A}}_{\left(k\right)}^{bb}$ are constructed as:

30 $${\mathbf{A}}_{\left(k\right)i,i}^{bb}={f}_{s}^{b}\left(1-{f}_{s}^{b}\right)+{f}_{d}^{b}\left(1-{f}_{d}^{b}\right)+\frac{1}{2}{A}_{\left(k\right)s,d}^{bb},$$

and

$${\mathbf{A}}_{(k)i,{i}^{{\prime}}}^{bb}=\frac{1}{2}\left({A}_{\left(k\right)s,{i}^{{\prime}}}^{bb}+{A}_{\left(k\right)d,{i}^{{\prime}}}^{bb}\right).$$

Matrices that describe the relationship of LBP effects between breeds, ${\mathbf{A}}_{\left(k\right)}^{b{b}^{{\prime}}}$, when $b\ne {b}^{{\prime}}$ are constructed as:

31 $${\mathbf{A}}_{\left(k\right)i,i}^{b{b}^{{\prime}}}={-f}_{s}^{b}{f}_{s}^{{b}^{{\prime}}}-{f}_{d}^{b}{f}_{d}^{{b}^{{\prime}}}+\frac{1}{2}{A}_{\left(k\right)s,d}^{b{b}^{{\prime}}},$$

and

$${\mathbf{A}}_{(k)i,{i}^{{\prime}}}^{b{b}^{{\prime}}}=\frac{1}{2}\left({A}_{\left(k\right)s,{i}^{{\prime}}}^{b{b}^{{\prime}}}+{A}_{\left(k\right)d,{i}^{{\prime}}}^{b{b}^{{\prime}}}\right).$$

Note that ${\mathbf{A}}_{(k)}^{b{b}^{{\prime}}}$ is a symmetric matrix and ${\mathbf{A}}_{(k)}^{b{b}^{{\prime}}}={\mathbf{A}}_{(k)}^{{b}^{{\prime}}b}$.

Based on these derivations, we propose a pedigree-based model that partitions the breeding value as an alternative to the model of Garcia-Cortes and Toro [6], in which we consider pedigree LBP relationship matrices, ${\mathbf{A}}_{(k)}^{b{b}^{{\prime}}}$, for ${n}_{b}-1$ breeds and their covariances instead of BS effects for each pair of breeds. The ${\widetilde{\mathbf{k}}}^{b}$ terms in Eq. (19) can be estimated from pedigree by assuming:

$$\left[\begin{array}{c}{\widetilde{\mathbf{k}}}^{1}\\ \vdots \\ {\widetilde{\mathbf{k}}}^{{n}_{b}-1}\end{array}\right]\sim N\left(\mathbf{0},\left[\begin{array}{ccc}{\mathbf{A}}_{(k)}^{11}{\sigma }_{k(11)}^{2}& \cdots & {\mathbf{A}}_{(k)}^{1,{n}_{b}-1}{\sigma }_{k(1, {n}_{b}-1)}^{2}\\ \vdots & \ddots & \vdots \\ {\mathbf{A}}_{(k)}^{1,{n}_{b}-1}{\sigma }_{k(1, {n}_{b}-1)}^{2}& \cdots & {\mathbf{A}}_{(k)}^{{n}_{b}-1,{n}_{b}-1}{\sigma }_{k({n}_{b}-1,{n}_{b}-1)}^{2}\end{array}\right]\right).$$

Similar to the genomic LBP (co)variance structure, the combined pedigree LBP (co)variance matrix cannot be directly expressed as a Kronecker product of the relationship matrix and the (co)variance component matrix. However, an alternative model can be formed by including artificial effects, as described for the genomic model in Eq. (21).

Garcia-Cortes and Toro [6] showed that a generalized inverse of the partial BS relationship matrices can be constructed based on a slight modification of the Quaas [17] procedure for constructing the inverse of the numerator relationship matrix. A generalized inverse of ${\mathbf{A}}_{(k)}^{bb}$ can be found in a similar manner. However, for models with more than two breeds, the inverse of the matrix $${\mathbf{A}}_{(k)}=\left[\begin{array}{ccc}{\mathbf{A}}_{(k)}^{11}& \cdots & {\mathbf{A}}_{(k)}^{1,{n}_{b}-1}\\ \vdots & \ddots & \vdots \\ {\mathbf{A}}_{(k)}^{1,{n}_{b}-1}& \cdots & {\mathbf{A}}_{(k)}^{{n}_{b}-1,{n}_{b}-1}\end{array}\right]$$ is needed, which has a more complicated structure.

### Converting (co)variances

The estimated LBP variance components may depend on the choice of the reference breed, ${b}^{*}$, and are not easily interpreted. However, with the derivations presented here, we can convert the estimated LBP (co)variances to represent the better-known concept of BS variance, as will be described in the following.

As detailed in Appendix 5, with some algebra on the expression for BS variance in Eq. (10) and for the LBP (co)variance in Eq. (18), the BS variance between breeds $b$ and ${b}^{{\prime}}$ is calculated from the LBP (co)variance as:

32 $${\sigma }_{w\left(b,{b}^{{\prime}}\right)}^{2}=\frac{1}{2}{\sigma }_{k(bb)}+\frac{1}{2}{\sigma }_{k({b}^{{\prime}}{b}^{{\prime}})}-{\sigma }_{k\left({bb}^{{\prime}}\right)},$$

and for the BS variance between the reference breed for LBP, ${b}^{*}$, and breed $b$ as:

$${\sigma }_{w\left(b,{b}^{*}\right)}^{2}=\frac{1}{2}\sum_{j=1}^{m}{\left({\epsilon }_{j}^{b}-{\epsilon }_{j}^{{b}^{*}}\right)}^{2}=\frac{1}{2}{\sigma }_{k(bb)}.$$

It follows that estimates of LBP variances can be obtained from estimated BS variances as ${\sigma }_{k(bb)}=2{\sigma }_{w\left(b,{b}^{*}\right)}^{2}$. Furthermore, the covariance between LBP effects for breeds $b$ and ${b}^{{\prime}}$ can be calculated from BS variances as (derivation in Appendix 5) ${\sigma }_{k({bb}^{{\prime}})}={\sigma }_{w\left(b,{b}^{*}\right)}^{2}+{\sigma }_{w\left({b}^{{\prime}},{b}^{*}\right)}^{2}-{\sigma }_{w\left(b,{b}^{{\prime}}\right)}^{2}$.

In the case of two breeds, $b$ and ${b}^{{\prime}}$, we only have a single BS term and a single LBP term. Furthermore, ${\mathbf{Q}}^{bb}{\sigma }_{k(bb)}={\mathbf{W}}^{b{,b}^{{\prime}}}{\sigma }_{w(b,{b}^{{\prime}})}^{2}$, ${\mathbf{Q}}^{bb}=2{\mathbf{W}}^{b,{b}^{{\prime}}}$, and ${\sigma }_{k(bb)}=\frac{1}{2}{\sigma }_{w(b,{b}^{{\prime}})}^{2}$.

### Example I

For a demonstration of the partial BS relationship and similarity matrices and the partial LBP relationship matrices, Table 1 presents the matrices for a small crossbred pedigree for three breeds, A, B, and C, with nine individuals. We constructed the pedigree-based BS relationship matrices for these individuals using Eq. (6). We also obtained genotypes and assigned BOA for three crossbred dairy cows, which had crossbred dams and the same pedigree structure as individuals 7 to 9 in Table 1. The genotypes are a part of the dataset used by Eiríksson et al. [18], where further details can be found. Based on these data, we constructed the ${\mathbf{T}}^{b}$ matrices, which were then used to construct the genomic BS similarity matrices using Eq. (9). We calculated the values of a genomic partial LBP relationship matrices for the same cows for breeds A and B and the genomic matrix describing the relationship of LBP effects between breeds A and B using Eq. (17). Furthermore, for individuals 7 to 9 in Table 1, we constructed the pedigree-based partial LBP relationship matrices for breeds A and B (setting breed C as the reference breed) using Eq. (30) and the matrix of relationships between LBP effects of breeds A and B using Eq. (31).

**Table 1:** Pedigree and breed proportions for Example I

Animal	Sire	Dam	Breed proportions		
			A	B	C
1			1	0	0
2			1	0	0
3			1	0	0
4			0	1	0
5			0	0	1
6	4	1	0.5	0.5	0
7	5	6	0.25	0.25	0.5
8	2	7	0.625	0.125	0.25
9	3	7	0.625	0.125	0.25

### Example II

To demonstrate and test a model with the genomic partial LBP relationship matrices, we simulated genotypes and phenotypes for crossbreds of three breeds using the QMSim software [19] (see Additional file 1 for the QMSim instruction code). We simulated a genome consisting of 10 chromosomes of 50 cM. Initially, each chromosome had 1000 evenly spaced biallelic markers and 50 randomly placed biallelic QTL. The QTL effects were randomly drawn from a gamma distribution with shape parameter 0.5. The simulated trait had a heritability of 0.3. Throughout the simulation, each female had five offspring with an equal probability of being male or female.

The 1000 historical generations consisted of 2000 animals each. From the last historical generation, three populations, A, B and C, were formed by randomly selecting 20 males and 20 females as founders of each population. Each population was then randomly mated for 40 discrete generations with replacements selected at random from the offspring of the previous generation. In the first 10 generations, the number of females in each population increased linearly to 60, but population size was kept stable thereafter. A single generation of F1 crossbred animals was formed by mating 20 randomly selected males from generation 39 of population A to 60 randomly selected females from generation 39 of population B. Subsequently, another crossbred population, D, was formed by mating 20 randomly selected males from generation 40 of population C to 100 randomly selected females from the F1 crossbred population. Population D mated randomly for three generations, resulting in 1500 animals with one or both parents being crossbred.

We used the marker genotypes of generations 38 to 40 of populations A, B and C, and all three generations of population D to test our model. For these genotypes, 7375 SNPs had a minor allele frequency higher than 0.05 and were retained for further analysis. In the output of QMSim, the phase of the genotypes is available in the output. However, for the crossbred individuals, the population of origin of the alleles is not in the output. Therefore, we assigned the marker alleles in the D population to BOA using the AllOr method [10] and using the phased genotypes from generations 38 to 40 of the purebred populations as reference haplotypes for AllOr. Furthermore, we set the window size for the BOA assignment to 50 SNPs with an overlap of 45 SNPs. Based on the assigned BOA and the genotypes of population D, we formed the within-breed genomic relationship matrices based on Eq. (5), with allele frequencies ${\mathbf{p}}^{b}$ calculated from generations 38 to 40 for populations $b=\text{A},\text{B},\text{C}$, and standardizing factor ${c}_{b}=\sum_{j=1}^{m}2{p}_{j}^{b}(1-{p}_{j}^{b})$. We set population C as reference breed. Thus, only LBP effects for breeds A and B were included in the model. The LBP relationship matrix was constructed as $${\mathbf{Q}}^{AB}=\left[\begin{array}{c}{\mathbf{Z}}^{A}\\ {\mathbf{Z}}^{B}\end{array}\right]{\left[\begin{array}{c}{\mathbf{Z}}^{A}\\ {\mathbf{Z}}^{B}\end{array}\right]}^{T}\times \frac{1}{m}$$, where ${\mathbf{Z}}^{A}$ and ${\mathbf{Z}}^{B}$ contain the BOA information and were calculated as described for ${\mathbf{Z}}^{b}$ in Eq. (7), and $m$ is the number of SNPs in the analysis (7375).

For the variance component estimation, we considered the following model:

$$\mathbf{y}=\mathbf{F}{\varvec{\updelta}}+{\mathbf{a}}_{A}+{\mathbf{a}}_{B}+{\mathbf{a}}_{C}+{\mathbf{X}}_{A}{\widetilde{\mathbf{k}}}_{\star }^{A}+{\mathbf{X}}_{B}{\widetilde{\mathbf{k}}}_{\star }^{B}+\mathbf{e},$$

where vector $\mathbf{y}$ contains the phenotypes of 1500 individuals from population D, matrix $\mathbf{F}$ contains the proportion of alleles assigned to each breed, vector ${\varvec{\updelta}}$ contains the effects of breed proportions, vectors ${\mathbf{a}}_{A}$, ${\mathbf{a}}_{B}$, and ${\mathbf{a}}_{C}$ contain the partial genetic effects for breeds A, B, and C, respectively, vector $${\widetilde{\mathbf{k}}}_{\star }^{A}=\left[\begin{array}{c}{\widetilde{\mathbf{k}}}^{A}\\ {\widetilde{\mathbf{k}}}_{\star }^{B,A}\end{array}\right]$$ contains the modified LBP effects of breed A (${\widetilde{\mathbf{k}}}^{A}$) and artificial effects ${\widetilde{\mathbf{k}}}_{\star }^{B,A}$, and matrix $${\mathbf{X}}_{A}=\left[\begin{array}{cc}\mathbf{I}& \mathbf{0}\end{array}\right]$$ connects the phenotypes to the first 1500 elements of ${\widetilde{\mathbf{k}}}_{\star }^{A}$. Similarly, $${\widetilde{\mathbf{k}}}_{\star }^{B}=\left[\begin{array}{c}{\widetilde{\mathbf{k}}}_{\star }^{A,B}\\ {\widetilde{\mathbf{k}}}^{B}\end{array}\right]$$ contains the modified LBP effects of breed B (${\widetilde{\mathbf{k}}}^{B}$) and artificial effects (${\widetilde{\mathbf{k}}}_{\star }^{A,B}$), and $${\mathbf{X}}_{B}=\left[\begin{array}{cc}\mathbf{0} & \mathbf{I}\end{array}\right]$$. We assumed the variance structure to be ${\mathbf{a}}_{b}\sim N(\mathbf{0},{\sigma }_{a(b)}^{2}{\mathbf{G}}^{b})$ for populations $b=$ A, B, and C, $$\left[\begin{array}{c}{\widetilde{\mathbf{k}}}_{\star }^{A}\\ {\widetilde{\mathbf{k}}}_{\star }^{B}\end{array}\right]\sim N(\mathbf{0},\left[\begin{array}{cc}{\sigma }_{k(AA)}& {\sigma }_{k(AB)}\\ {\sigma }_{k(AB)}& {\sigma }_{k,(BB)}\end{array}\right]\otimes {\mathbf{Q}}^{AB})$$, and $\mathbf{e}\sim N(\mathbf{0},{\sigma }_{e}^{2}\mathbf{I})$, where ${\sigma }_{e}^{2}$ is the residual variance.

We estimated the variance components using the average information REML (AI-REML) algorithm implemented in the DMU package [20]. We calculated the inverses of the relationship matrices ${\mathbf{G}}^{A}$, ${\mathbf{G}}^{B}$, ${\mathbf{G}}^{C}$ and ${\mathbf{Q}}^{AB}$ using Julia [21] (see Additional file 2). We added a small value, 0.0001, to the diagonal of the relationship matrices to avoid singularity.

For comparison with the estimated values, we calculated the true variance components based on the simulated QTL effects and allele frequencies. The within-population additive genetic variance for each population $b$ was calculated as ${{\sigma }_{a\left(b\right)}^{2}}_{true}=2\sum_{j}{{p}_{QTL,b}}_{j}(1-{{p}_{QTL,b}}_{j}){({\beta }_{j,1}-{\beta }_{j,2})}^{2}$, where the summation is over all segregating QTL, ${{p}_{QTL,b}}_{j}$ is the frequency of allele 1 for QTL $j$ in population $b$, calculated from generations 38 to 40, and ${\beta }_{j,1}$ (${\beta }_{j,2}$) is the simulated effect of allele 1 (2) for QTL $j$. The true LBP variances were calculated as ${{\sigma }_{k\left(b{b}^{{\prime}}\right)}^{2}}_{true}=\sum_{j}({\epsilon }_{j}^{b}-{\epsilon }_{j}^{C})({\epsilon }_{j}^{{b}^{{\prime}}}-{\epsilon }_{j}^{C})$ for $b=A,B$ and ${b}^{{\prime}}=A,B$, and ${\epsilon }_{j}^{b}={{p}_{QTL,b}}_{j}{\beta }_{j,1}+\left(1-{{p}_{QTL,b}}_{j}\right){\beta }_{j,2}$ for $b=\text{A},\text{B},\text{C}$.

## Results

### Example I

The pedigree-based partial BS relationship matrices for individuals 7, 8, and 9, which are those with non-zero elements in at least one BS matrix, are in Table 2. For individual 7, which is the offspring of a F1 crossbred dam of breeds A and B, and a purebred sire of breed C, BS is only expected between breeds A and B and therefore all elements related to this individual in the pedigree-based partial relationship matrices for BS between A and C, and B and C, are 0. The genomic BS similarity matrices are in Table 3. The values in the genomic BS similarity matrices deviate from those in the pedigree-based matrices but show the same pattern. For segregation between breeds A and C and between breeds B and C, the diagonal element for individual 7 is 0, while the off-diagonal elements that connect individual 7 to individuals 8 and 9 are non-zero. These matrices are, therefore, not valid relationship matrices.

**Table 2:** Pedigree-based breed-segregation relationship matrices for Example 1

Breeds	Animal	7	8	9
A-B	7	0.5	0.25	0.25
	8	0.25	0.125	0.125
	9	0.25	0.125	0.125
A-C	7	0	0	0
	8	0	0.25	0
	9	0	0	0.25
B-C	7	0	0	0
	8	0	0.25	0
	9	0	0	0.25

The coefficients from pedigree-based partial breed-segregation relationship matrices for animals with a crossbred parent in the pedigree in Table 1

**Table 3:** Genomic breed-segregation similarity matrices for Example 1

Breeds	Animal	7	8	9
A-B	7	0.48	0.25	0.18
	8	0.25	0.12	0.09
	9	0.18	0.09	0.07
A-C	7	0.00	0.03	− 0.02
	8	0.03	0.25	0.06
	9	− 0.02	0.06	0.19
B-C	7	0.00	− 0.03	0.02
	8	− 0.03	0.25	− 0.06
	9	0.02	− 0.06	0.28

The coefficients from genomic breed segregation similarity matrices for animals with a crossbred parent in the pedigree in Table 1

Table 4 presents the pedigree-based partial LBP relationship matrices for breeds A and B for individuals 7, 8, and 9, as well as the matrix describing the relationship between the LBP effects of breeds A and B. In this example, the matrices for breeds A and B are identical. All elements of the between-breed partial LBP relationship matrix (A-B) are negative. The genomic partial LBP relationship matrices are in Table 5. Similar to the comparison between the genomic and pedigree BS matrices, the elements of the genomic LBP partial relationship matrices deviate from those in the pedigree matrices but show the same pattern. The genomic matrix with relationships of LBP effects between breeds is non-symmetric, in contrast to its pedigree-based counterpart.

**Table 4:** Pedigree-based local breed proportion relationship matrices for Example 1

Breed	Animal	7	8	9
A	7	0.25	0.125	0.125
	8	0.125	0.1875	0.0625
	9	0.125	0.0625	0.1875
B	7	0.25	0.125	0.125
	8	0.125	0.1875	0.0625
	9	0.125	0.0625	0.1875
A-B	7	− 0.25	− 0.125	− 0.125
	8	− 0.125	− 0.0625	− 0.0625
	9	− 0.125	− 0.0625	− 0.0625

The coefficients from the pedigree-based partial local breed proportion relationship matrices for breeds A and B and the elements from the pedigree-based matrix describing the relationship of local breed proportion effects between breeds A and B. Values are shown for animals with a crossbred parent in the pedigree in Table 1. Breed C is the reference breed

**Table 5:** Genomic local breed proportion matrices for Example 1

Breed	Animal	7	8	9
A	7	0.24	0.14	0.08
	8	0.14	0.19	0.08
	9	0.08	0.08	0.13
B	7	0.24	0.11	0.10
	8	0.11	0.19	0.02
	9	0.10	0.02	0.17
A-B	7	− 0.24	− 0.14	− 0.08
	8	− 0.11	− 0.06	− 0.04
	9	− 0.10	− 0.06	− 0.03

The coefficients from the genomic local breed proportion partial relationship matrices for breeds A and B and the elements from the genomic matrix describing the relationship of local breed proportion effects between breeds A and B. Values are shown for animals with a crossbred parent in the pedigree in Table 1. Breed C is the reference breed

### Example II

The AI-REML algorithm converged in 10 rounds of iterations. The true and estimated variance components from Example II are presented in Table 6. The within-breed additive genetic variances were slightly smaller than the simulated values for the last historical generation. This reduction in genetic variance is expected because of the small population sizes for the 40 generations that separated the populations, resulting in considerable genetic drift. This genetic drift, however, resulted in substantial segregation between the populations, as reflected in the true LBP variance of up to 0.10 (Population B; Table 6). The true BS variances were 0.04, 0.03, and 0.05 for ${\sigma }_{w\left(A,B\right)}^{2}$, ${\sigma }_{w\left(A,C\right)}^{2}$, and ${\sigma }_{w\left(B,C\right)}^{2}$, respectively. The estimated LBP variances were on the same level as their true values, while the estimated population-specific additive genetic variances were slightly smaller than their true values.

**Table 6:** Estimated and true variance components for the simulated data from Example II

	${\sigma }_{a(A)}^{2}$	${\sigma }_{a(B)}^{2}$	${\sigma }_{a(C)}^{2}$	${\sigma }_{k(AA)}$	${\sigma }_{k(BB)}$	${\sigma }_{k(AB)}$	${\sigma }_{e}^{2}$
True	0.26	0.27	0.23	0.07	0.10	0.04	0.70
Estimated	0.23 ± 0.09	0.21 ± 0.08	0.19 ± 0.05	0.06 ± 0.04	0.11 ± 0.06	0.01 ± 0.04	0.75 ± 0.03

Breed specific additive genetic variance for breed A (${\sigma }_{a(A)}^{2}$), B (${\sigma }_{a(B)}^{2}$), and C (${\sigma }_{a(C)}^{2}$), and local breed proportion variance for breed A (${\sigma }_{k(AA)}$) and B (${\sigma }_{k(BB)}$), local breed proportion covariance between breed A and B (${\sigma }_{k(AB)}$), and residual variance (${\sigma }_{e}^{2}$), with standard errors of the estimates

## Discussion

In this paper, we present the theoretical background for including LBP effects in genetic and genomic evaluation models for crossbred populations with varying breed compositions based on pedigree information or BOA of SNP genotypes. We also show that LBP and BS account for the same extra additive genetic variance in later generations of crossbreeding compared to the first generation. Furthermore, we provide a method for constructing the genomic BS similarity matrices based on the estimated BOA of genotypes for a general crossbreeding population. However, we found that the genomic BS similarity matrices are not necessarily positive semidefinite and present an alternative method that consists of setting one breed as reference breed and fitting LBP effects of all other breeds, while accounting for the correlation between the LBP effects of different breeds. This model was applied to a simulated dataset.

The results from Example I illustrate the properties of the relationship matrices for a small toy example. Interestingly, the genomic BS similarity matrix between breeds A and B (Table 3) was not positive semi-definite, underlining the limitations of modelling BS using BOA information. The results from Example II demonstrate the ability of the genomic LBP model to capture the effects of segregation between breeds in crossbred populations. The simulated scenario was intentionally designed to obtain large LBP variances, with multiple generations since the separation of the breeds, a small size of the purebred populations, and relatively few (500) QTL affecting the trait, and this was evident from the results. Furthermore, the crossbreeding structure with both parents of all individuals in generations 2 and 3 of population D being crossbred, facilitated estimation of LBP effects. In addition, the half-sib and full-sib progeny from crossbred parents are expected to increase the LBP relationships between individuals. Still, the fast convergence of the AI-REML algorithm and variance estimates that were close to their true variances, for a data set with phenotypes of only 1500 individuals, supports that the model will be able to disentangle the contribution of breed-specific and LBP effects to the phenotypes of crossbreds in real data sets also.

The models proposed in this paper are relevant for genetic and genomic prediction based on data from more than one generation of crossbreeding, for which the breeding values of crossbreds are partitioned into breed-specific terms [6]. Previous studies [11–13] have presented BOA models with BS terms for specific cases only [11, 12]. The BS terms presented in the supplementary material of Aase et al. [13] are equivalent to our genomic BOA model with BS, and thus, suffer from the same problem of matrices that may be not positive semi-definite. The way we propose to account for segregation between breeds, i.e. with LBP effects, can be applied to any number of breeds or breed composition and is, therefore, more general than the methods presented by Christensen et al. [11] and Rio et al. [12].

Compared to the pedigree-based model of García-Cortés and Toro [6], for the models with LBP effects, a complication for estimation of variance components is that the LBP (co)variance structure cannot be directly expressed as a Kronecker product. Therefore, the model must be extended with artificial effects (Eq. 21) for variance component estimation. In Example II, we successfully estimated the variance components using AI-REML implemented in a commonly used mixed model software for genetic evaluation, using such an extension. Therefore, the need to extend the models with artificial effects appears to be only a minor complication. When the variance components are assumed to be known and only the effects need to be predicted, the extension can be avoided by multiplying the parts of ${\mathbf{Q}}^{b,{b}^{{\prime}}}$ relating to each breed, or to each pair of breeds, with the appropriate (co)variance estimates, as shown in Eq. (20).

In a terminal crossbreeding system, the crossbred animals are not potential breeding stock and estimates of the ${\widetilde{\mathbf{k}}}^{b}$ or ${\mathbf{w}}^{b,{b}^{{\prime}}}$ terms will therefore not be a part of the predicted breeding values required for selection, but they are used only to reduce the residual error when including records on crossbreds to improve the prediction for purebred selection candidates. Thus, ignoring BS or LBP, as was done in the literature [8, 16, 22, 23], is unlikely to have a considerable impact on selection among purebred selection candidates, even if there was significant BS. When there is selection among crossbred animals, which can occur in, e.g. rotational crossbreeding systems, the BS terms are part of the breeding values of the crossbred individuals. In this case, models with or without a BS term could lead to different selection decisions. However, models for genomic evaluation in a rotational crossbreeding system of three breeds have excluded BS and LBP effects in previous studies on simulated data [12, 13]. The same is true for studies with BOA models applied to real data for admixed populations [24] or populations with crossbreeding involving varying breed combinations [18]. For such scenarios, the LBP model presented here could improve predictions of breeding values if there was significant BS, which would need investigation. Although the focus of this paper is on segregation between breeds in a livestock context, the developed models may also be applicable to model segregation between genetic groups in plant breeding [12].

For the pedigree-based model, we have not found a simple algorithm for the construction of the generalized inverses of the partial LBP relationship matrices when covariances between LBP effects for different breeds are included. Therefore, inclusion of BS effects, as in García-Cortés and Toro [6], is more attractive for pedigree-based models than for the pedigree-based LBP model. However, for single-step genomic models [2, 3], phenotypes for both genotyped and non-genotyped individuals are included. For such models, compatible pedigree and genomic relationship matrices are important [25]. Christensen et al. [11] presented a model for three-way terminal crossbreeding with a combined genomic and pedigree-based partial BS relationship matrix. However, for more complex crossbreeding scenarios, the genomic BS similarity matrix may not be positive semi-definite. Therefore, single-step models for complex crossbreeding should include LBP effects rather than BS and, thus, pedigree-based LBP matrices are needed. Values for the pedigree and genomic partial LBP relationship matrices presented here have the same expectation if pedigree information is complete, i.e. when the pedigree of all crossbred individuals can be traced back to purebred ancestors and BOA assignment is complete, i.e. all alleles are assigned breed origin. Compatibility of the partial LBP matrices is demonstrated in the results of Example I, Tables 4 and 5. The genomic and pedigree partial LBP relationship matrices can therefore be combined in a similar manner as in Christensen et al. [11], but single-step models with LBP effects need further investigation.

For a simulated data set in a two-breed rotational crossbreeding system, Poulsen et al. [26] tested different relationship matrices and found that partitioning the breeding value as García-Cortés and Toro [6] performed similar to the metafounder approach [27] and outperformed other options. Both these approaches include BS. No genotypes of crossbreds were included in the study by Poulsen et al. [26]. Further comparison of models for rotational crossbreeding could include genotyped crossbreds and account for BS or LBP effects using the methods presented in this paper.

For practical genetic and genomic evaluation, the relevance of LBP or BS terms depends on the magnitude of the LBP or BS variance in the population analysed. Such estimates are rare in the literature. Eiríksson et al. [14] found small but statistically significant LBP variance for milk production traits in crossbred dairy cows. Munilla Leguizamón and Cantet [28] did not find significant BS variance for weaning weight in Angus × Hereford crossbred beef cattle. However, Birchmeier et al. [29] found significant BS variance for birth weight in crossbred beef cattle. Here, we have shown that LBP effects can be modelled based on assigned BOA in genomic models for data with complicated crossbreeding structures. Although BOA assignment is generally not complete and contains errors [10, 18, 30], BOA information should provide a more precise estimate of LBP or BS relationships than pedigree-based models. Therefore, the models developed here should facilitate more accurate estimation of BS or LBP variances than the previous pedigree-based models and open the possibility of accounting for BS or LBP in genomic predictions for crossbred populations with BOA models, regardless of the crossbreeding structure.

Based on the results presented here, combined with previous work on the inclusion of BS effects in genetic models, we recommend that for pedigree-based models, BS terms are included following García-Cortés and Toro [6]. For genomic BOA models applied to data that include segregation for two breeds only, either LBP or BS effects can be included. However, for genomic BOA models applied to data that includes segregation for more than one pair of breeds, LBP terms should be used, as presented here, rather than BS terms.

## Conclusions

Models for the genetic evaluation using crossbred data that partition the genetic value into breed-specific terms should, in theory, account for the effects of LBP or BS. The (co)variance structure for BS effects in genomic BOA models for crossbred data involving more than two breeds are not guaranteed to lead to relationship matrices that are positive semi-definite. The LBP (co)variance structure can be constructed from the pedigree or BOA information by including LBP effects for each breed except for a reference breed, given that the covariance between LBP effects of each pair of breeds is included in the model.

### Supplementary Information

**Additional file 1.** It contains the QMSim instruction file for the simulation in Example II.

**Additional file 2.** It contains the Julia code for the construction and inversion of the relationship matrices used in Example II.

## Data Availability

Data and scripts from Example II are available from the first author upon reasonable request.

## Appendix 1

Here, we derive the formulas for breed-segregation (BS) variance and covariance for a single locus in Eqs. (7) and (8), respectively. The contribution of locus $j$ to the BS variance of individual $i$, a crossbred of ${n}_{b}$ breeds, is $Var\left(\sum_{b}{\epsilon }_{j}^{b}{\ddot{z}}_{{i}^{j}}^{b}\right)=E\left[{\left(\sum_{b}{\epsilon }_{j}^{b}{\ddot{z}}_{{i}^{j}}^{b}\right)}^{2}\right]$. Furthermore,

$$\left( { \sum \limits_{b} \epsilon_{j}^{b} \ddot{z}_{{i^{j} }}^{b} } \right)^{2} = \sum \limits_{b} {\epsilon_{j}^{b}}^{2} {{\ddot{z}_{{i^{j}}}^{b}}}{^{2}} + \sum \limits_{b} \sum \limits_{{b^{\prime} > b}} 2\epsilon_{j}^{b} \epsilon_{j}^{{b^{\prime}}} \ddot{z}_{{i^{j} }}^{b} \ddot{z}_{{i^{j} }}^{{b^{\prime}}} .$$

Using $\sum_{b}{\ddot{z}}_{{i}^{j}}^{b}=0$, we get ${\left(\sum_{b}{\epsilon }_{j}^{b}{\ddot{z}}_{{i}^{j}}^{b}\right)}^{2}=\sum_{b}\sum_{{b}^{{\prime}}\ne b}-{{\epsilon }_{j}^{b}}^{2}{\ddot{z}}_{{i}^{j}}^{b}{\ddot{z}}_{{i}^{j}}^{{b}^{{\prime}}}+\sum_{b}\sum_{{b}^{{\prime}}>b}2{\epsilon }_{j}^{b}{\epsilon }_{j}^{{b}^{{\prime}}}{\ddot{z}}_{{i}^{j}}^{b}{\ddot{z}}_{{i}^{j}}^{{b}^{{\prime}}}$.

Rearranging, gives:

$$\begin{aligned} \left( { \sum \limits_{b} \epsilon_{j}^{b} \ddot{z}_{{i^{j} }}^{b} } \right)^{2} & = \sum \limits_{b} \left( { \sum \limits_{{b^{\prime} > b}} - {\epsilon_{j}^{b}}^2 \ddot{z}_{{i^{j} }}^{b} \ddot{z}_{{i^{j} }}^{{b^{\prime}}} + \sum \limits_{{b^{\prime} > b}} - {\epsilon_{j}^{b^{\prime}}}^{2} \ddot{z}_{{i^{j} }}^{b} \ddot{z}_{{i^{j} }}^{{b^{\prime}}} \sum \limits_{{b^{\prime} > b}} 2\epsilon_{j}^{b} \epsilon_{j}^{{b^{\prime}}} \ddot{z}_{{i^{j} }}^{b} \ddot{z}_{{i^{j} }}^{{b^{\prime}}} } \right) \\ & = \sum \limits_{b} \sum \limits_{{b^{\prime} > b}} - \ddot{z}_{{i^{j} }}^{b} \ddot{z}_{{i^{j} }}^{{b^{\prime}}} \left( {{\epsilon_{j}^{b}}^2 - 2\epsilon_{j}^{b} \epsilon_{j}^{{b^{\prime}}} + {\epsilon_{j}^{b^{\prime}}}^2 } \right) = \sum \limits_{b} \sum \limits_{{b^{\prime} > b}} - \ddot{z}_{{i^{j} }}^{b} \ddot{z}_{{i^{j} }}^{{b^{\prime}}} \left( {\epsilon_{j}^{b} - \epsilon_{j}^{{b^{\prime}}} } \right)^{2} . \\ \end{aligned}$$

Therefore, $$Var\left(\sum_{b}{\epsilon }_{j}^{b}{\ddot{z}}_{{i}^{j}}^{b}\right)=\sum_{b}\sum_{{b}^{{\prime}}>b}-{\ddot{z}}_{{i}^{j}}^{b}{\ddot{z}}_{{i}^{j}}^{{b}^{{\prime}}}E\left[{\left({\epsilon }_{j}^{b}-{\epsilon }_{j}^{{b}^{{\prime}}}\right)}^{2}\right]$$.

The contribution of locus $j$ to the BS covariance between animals $i$ and ${i}^{{\prime}}$ is: $Cov\left(\sum_{b}{\epsilon }_{j}^{b}{\ddot{z}}_{{i}^{j}}^{b},\sum_{b}{\epsilon }_{j}^{b}{\ddot{z}}_{{{i}^{{\prime}}}^{j}}^{b}\right)=E\left[\left(\sum_{b}{\epsilon }_{j}^{b}{\ddot{z}}_{{i}^{j}}^{b}\right)\left(\sum_{b}{\epsilon }_{j}^{b}{\ddot{z}}_{{{i}^{{\prime}}}^{j}}^{b}\right)\right]$. Furthermore, $\left(\sum_{b}{\epsilon }_{j}^{b}{\ddot{z}}_{{i}^{j}}^{b}\right)\left(\sum_{b}{\epsilon }_{j}^{b}{\ddot{z}}_{{{i}^{{\prime}}}^{j}}^{b}\right)=\sum_{b}{{\epsilon }_{j}^{b}}^{2}{\ddot{z}}_{{i}^{j}}^{b}{\ddot{z}}_{{{i}^{{\prime}}}^{j}}^{b}+\sum_{b}\sum_{{b}^{{\prime}}>b}{\epsilon }_{j}^{b}{\epsilon }_{j}^{{b}^{{\prime}}}{\ddot{z}}_{{i}^{j}}^{b}{\ddot{z}}_{{{i}^{{\prime}}}^{j}}^{{b}^{{\prime}}}+\sum_{b}\sum_{{b}^{{\prime}}>b}{\epsilon }_{j}^{b}{\epsilon }_{j}^{{b}^{{\prime}}}{\ddot{z}}_{{{i}^{{\prime}}}^{j}}^{b}{\ddot{z}}_{{i}^{j}}^{{b}^{{\prime}}}$.

Substituting ${{\epsilon }_{j}^{b}}^{2}{\ddot{z}}_{{i}^{j}}^{b}{\ddot{z}}_{{{i}^{{\prime}}}^{j}}^{b}$ with $\sum_{{b}^{{\prime}} \ne b}-{{\epsilon }_{j}^{b}}^{2}{\ddot{z}}_{{i}^{j}}^{b}{\ddot{z}}_{{{i}^{{\prime}}}^{j}}^{{b}^{{\prime}}}$ and rearranging,

$$\begin{aligned} \left( { \sum \limits_{b} \epsilon_{j}^{b} \ddot{z}_{{i^{j} }}^{b} } \right)\left( {\sum \limits_{b} \epsilon_{j}^{b} \ddot{z}_{{i^{\prime{j}}}}^{b} } \right) & = \sum \limits_{b} \sum \limits_{{b^{\prime} \ne b}} - {\epsilon_{j}^{b}}^{2} \ddot{z}_{{i^{j} }}^{b} \ddot{z}_{{i^{\prime{j}}}}^{{b^{\prime}}} + \sum \limits_{b} \sum \limits_{{b^{\prime} > b}} \epsilon_{j}^{b} \epsilon_{j}^{{b^{\prime}}} \ddot{z}_{{i^{j} }}^{b} \ddot{z}_{{i^{\prime{j}}}}^{{b^{\prime}}} + \sum \limits_{b} \sum \limits_{{b^{\prime} > b}} \epsilon_{j}^{b} \epsilon_{j}^{{b^{\prime}}} \ddot{z}_{{i^{\prime{j}}}}^{b} \ddot{z}_{{i^{j} }}^{{b^{\prime}}} \\ & = \sum \limits_{b} \left( { \sum \limits_{{b^{\prime} > b}} - {\epsilon_{j}^{b}}^{2} \ddot{z}_{{i^{j} }}^{b} \ddot{z}_{{i^{\prime{j}}}}^{{b^{\prime}}} + \sum \limits_{{b^{\prime} > b}} - {\epsilon_{j}^{b^{\prime}}}^2 \ddot{z}_{{i^{j} }}^{b} \ddot{z}_{{i^{\prime{j}}}}^{{b^{\prime}}} + \sum \limits_{{b^{\prime} > b}} \epsilon_{j}^{b} \epsilon_{j}^{{b^{\prime}}} \ddot{z}_{{i^{j} }}^{b} \ddot{z}_{{i^{\prime{j}}}}^{{b^{\prime}}} + \sum \limits_{{b^{\prime} > b}} \epsilon_{j}^{b} \epsilon_{j}^{{b^{\prime}}} \ddot{z}_{{i^{j} }}^{{b^{\prime}}} \ddot{z}_{{i^{\prime{j}}}}^{b} } \right). \\ \end{aligned}$$

Furthermore,

$$\begin{aligned} \left( { \sum \limits_{b} \epsilon_{j}^{b} \ddot{z}_{{i^{j} }}^{b} } \right)\left( { \sum \limits_{b} \epsilon_{j}^{b} \ddot{z}_{{i^{\prime}}}^{b} } \right) & = \sum \limits_{b} \sum \limits_{{b^{\prime} > b}} - \frac{1}{2}\ddot{z}_{{i^{j} }}^{b} \ddot{z}_{{i^{\prime}}}^{{b^{\prime}}} \left( {{\epsilon_{j}^{b}}^{2} - 2\epsilon_{j}^{b} \epsilon_{j}^{{b^{\prime}}} + {\epsilon_{j}^{b^{\prime}}}^2} \right) + \sum \limits_{b} \sum \limits_{{b^{\prime} > b}} - \frac{1}{2}\ddot{z}_{{i^{j} }}^{{b^{\prime}}} \ddot{z}_{{i^{\prime}}}^{b} \left( {{\epsilon_{j}^{b}}^{2} - 2\epsilon_{j}^{b} \epsilon_{j}^{{b^{\prime}}} + {\epsilon_{j}^{b^{\prime}}}^2 } \right) \\ & = \sum \limits_{b} \sum \limits_{{b^{\prime} > b}} \left( { - \ddot{z}_{{i^{j} }}^{b} \ddot{z}_{{i^{\prime}}}^{{b^{\prime}}} - \ddot{z}_{{i^{j} }}^{{b^{\prime}}} \ddot{z}_{{i^{\prime}}}^{b} } \right)\frac{1}{2}\left( {\epsilon_{j}^{b} - \epsilon_{j}^{{b^{\prime}}} } \right)^{2} . \\ \end{aligned}$$

Therefore, $Cov\left(\sum_{b}{\epsilon }_{j}^{b}{\ddot{z}}_{{i}^{j}}^{b},\sum_{b}{\epsilon }_{j}^{b}{\ddot{z}}_{{{i}^{{\prime}}}^{j}}^{b}\right)=\sum_{b}\sum_{{b}^{{\prime}}>b}\left(-{\ddot{z}}_{{i}^{j}}^{b}{\ddot{z}}_{{{i}^{{\prime}}}^{j}}^{{b}^{{\prime}}}-{\ddot{z}}_{{i}^{j}}^{{b}^{{\prime}}}{\ddot{z}}_{{{i}^{{\prime}}}^{j}}^{b}\right)\frac{1}{2}E\left[{\left({\epsilon }_{j}^{b}-{\epsilon }_{j}^{{b}^{{\prime}}}\right)}^{2}\right]$, which gives the expression in Eq. (8) in the main text.

## Appendix 2

In Appendix 2, we investigate the modelling of three independent LBP effects in relation to the presented theory of BS. We assume that the marker loci are the QTL for simplicity, the origin of the alleles is known, and ${\epsilon }_{j}^{b}$ are random unknown variables with mean zero.

We name the three breeds as A, B and C. The genomic LBP variance for three breeds related to locus $j$ for animal $i$ is:

$$\text{Var}\left({\ddot{\kappa }}_{{i}^{j}}^{sum}\right)=E\left[{({\epsilon }_{j}^{A}{\ddot{z}}_{{i}^{j}}^{A}+{\epsilon }_{j}^{B}{\ddot{z}}_{{i}^{j}}^{B}+{\epsilon }_{j}^{C}{\ddot{z}}_{{i}^{j}}^{C})}^{2}\right]$$. Furthermore,

33 $$\begin{aligned} \left( {\epsilon_{j}^{A} \ddot{z}_{{i^{j} }}^{A} + \epsilon_{j}^{B} \ddot{z}_{{i^{j} }}^{B} + \epsilon_{j}^{C} \ddot{z}_{{i^{j}}}^{C} } \right)^{2} & = {\epsilon_{j}^{A}}^2 {{\ddot{z}_{i^{j}}^{A^2}}} + {\epsilon_{j}^{B^{2}}} {\ddot{z}_{{i^{j} }}^{B^{2}}} + {\epsilon_{j}^{C^2}} {\ddot{z}_{{i^{j} }}^{C^{2}}} \\ & \quad + 2\epsilon_{j}^{A} \epsilon_{j}^{B} \ddot{z}_{{i^{j} }}^{A} \ddot{z}_{{i^{j} }}^{B} + 2\epsilon_{j}^{A} \epsilon_{j}^{C} \ddot{z}_{{i^{j} }}^{A} \ddot{z}_{{i^{j} }}^{C} + 2\epsilon_{j}^{B} \epsilon_{j}^{C} \ddot{z}_{{i^{j} }}^{B} \ddot{z}_{{i^{j} }}^{C} . \\ \end{aligned}$$

First, we show that when ${z}^{A}+{z}^{B}+{z}^{C}=0$,

34 $$2{z}^{A}{z}^{B}={{z}^{C}}^{2}-{{z}^{A}}^{2}-{{z}^{B}}^{2}.$$

The proof is as follows. Note that ${z}^{A}=-{z}^{B}-{z}^{C}$ and ${z}^{B}=-{z}^{A}-{z}^{C}$. This leads to $2{z}^{A}{z}^{B}={z}^{A}\left(-{z}^{A}-{z}^{C}\right)+{z}^{B}\left(-{z}^{B}-{z}^{C}\right).$ Rearranging gives $2{z}^{A}{z}^{B}=-{{z}^{A}}^{2}-{{z}^{B}}^{2}-{z}^{C}\left({z}^{A}+{z}^{B}\right)={{z}^{C}}^{2}-{{z}^{A}}^{2}-{{z}^{B}}^{2}$ which completes the proof.

Substituting $2{z}^{A}{z}^{B}$ with ${{z}^{C}}^{2}-{{z}^{A}}^{2}-{{z}^{B}}^{2}$ and similarly with the other pairs of breeds in Eq. (33) gives:

$$\begin{aligned} \left( {\epsilon_{j}^{A} \ddot{z}_{{i^{j} }}^{A} + \epsilon_{j}^{B} \ddot{z}_{{i^{j} }}^{B} + \epsilon_{j}^{C} \ddot{z}_{{i^{j} }}^{C} } \right)^{2} & = {\epsilon_{j}^{A^2}} {\ddot{z}_{{i^{j} }}^{A^2}} + {\epsilon_{j}^{B^2}} {\ddot{z}_{{i^{j} }}^{B^2}} + {\epsilon_{j}^{C^2}} {\ddot{z}_{{i^{j} }}^{C^2}} + \epsilon_{j}^{A} \epsilon_{j}^{B} {\ddot{z}_{{i^{j} }}^{C^2}} \\ & \quad + \epsilon_{j}^{A} \epsilon_{j}^{C} {\ddot{z}_{{i^{j} }}^{B^2}} + \epsilon_{j}^{B} \epsilon_{j}^{C} {\ddot{z}_{{i^{j} }}^{A^2}} - \epsilon_{j}^{A} \epsilon_{j}^{B} {\ddot{z}_{{i^{j} }}^{A^2}} - \epsilon_{j}^{A} \epsilon_{j}^{C} {\ddot{z}_{{i^{j} }}^{A^2}} \\ & \quad - \epsilon_{j}^{B} \epsilon_{j}^{A} {\ddot{z}_{{i^{j} }}^{B^2}} - \epsilon_{j}^{B} \epsilon_{j}^{C} {\ddot{z}_{{i^{j} }}^{B^2}} - \epsilon_{j}^{C} \epsilon_{j}^{A} {\ddot{z}_{{i^{j} }}^{C^2}} - \epsilon_{j}^{C} \epsilon_{j}^{B} {\ddot{z}_{{i^{j} }}^{C^2}} . \\ \end{aligned}$$

Some rearranging gives:

$$\begin{aligned} \left( {\epsilon_{j}^{A} \ddot{z}_{{i^{j} }}^{A} + \epsilon_{j}^{B} \ddot{z}_{{i^{j} }}^{B} + \epsilon_{j}^{C} \ddot{z}_{{i^{j} }}^{C} } \right)^{2} & = {\ddot{z}_{{i^{j} }}^{A^2}} \left( {\epsilon_{j}^{A} - \epsilon_{j}^{B} } \right)\left( {\epsilon_{j}^{A} - \epsilon_{j}^{C} } \right) \\ & \quad + {\ddot{z}_{{i^{j} }}^{B^2}} \left( {\epsilon_{j}^{B} - \epsilon_{j}^{A} } \right)\left( {\epsilon_{j}^{B} - \epsilon_{j}^{C} } \right) + {\ddot{z}_{{i^{j} }}^{C^2}} \left( {\epsilon_{j}^{C} - \epsilon_{j}^{A} } \right)\left( {\epsilon_{j}^{C} - \epsilon_{j}^{B} } \right). \\ \end{aligned}$$

Therefore,

$$\begin{aligned} {\text{Var}}\left( {\ddot{\kappa }_{{i^{j} }}^{sum} } \right) & = {\ddot{z}_{{i^{j} }}^{A^2}} E\left[ {\left( {\epsilon_{j}^{A} - \epsilon_{j}^{B} } \right)\left( {\epsilon_{j}^{A} - \epsilon_{j}^{C} } \right)} \right] \\ & \quad + {\ddot{z}_{{i^{j} }}^{B^2}} E\left[ {\left( {\epsilon_{j}^{B} - \epsilon_{j}^{A} } \right)\left( {\epsilon_{j}^{B} - \epsilon_{j}^{C} } \right)} \right] + {\ddot{z}_{{i^{j} }}^{C^2}} E\left[ {\left( {\epsilon_{j}^{C} - \epsilon_{j}^{A} } \right)\left( {\epsilon_{j}^{C} - \epsilon_{j}^{B} } \right)} \right]. \\ \end{aligned}$$

This decomposition of $\text{Var}\left({\ddot{\kappa }}_{{i}^{j}}^{sum}\right)$ gives a sum over three independent LBP terms and could be extended to a relationship matrix across multiple individuals. However, the $E\left[\left({\epsilon }_{j}^{A}-{\epsilon }_{j}^{B}\right)\left({\epsilon }_{j}^{A}-{\epsilon }_{j}^{C}\right)\right]$ term is not guaranteed to be positive. Therefore, the variance for the whole genome, $\sum_{j}E\left[\left({\epsilon }_{j}^{A}-{\epsilon }_{j}^{B}\right)\left({\epsilon }_{j}^{A}-{\epsilon }_{j}^{C}\right)\right]$ is not necessarily non-negative, and therefore not a valid variance term, and the same holds for other combinations of breeds.

## Appendix 3

Here, we present the derivation of Eq. (11). The variance for LBP over all breeds $b$ for locus $j$ is:

$$Var\left({\ddot{\kappa }}_{{i}^{j}}^{sum}\right)=E\left[{\left(\sum_{b}{{\ddot{z}}_{{i}^{j}}^{b}\epsilon }_{j}^{b}\right)}^{2}\right].$$

We set one breed as reference breed, ${b}^{*}$. Next, for breeds $b\ne {b}^{*}$, and ${b}^{{\prime}}\ne {b}^{*}$, we substitute ${\epsilon }_{j}^{b}={\epsilon }_{j}^{{b}^{*}}+{\epsilon }_{j}^{b}-{\epsilon }_{j}^{{b}^{*}}$ and ${\epsilon }_{j}^{{b}^{{\prime}}}={\epsilon }_{j}^{{b}^{*}}+{\epsilon }_{j}^{{b}^{{\prime}}}-{\epsilon }_{j}^{{b}^{*}}$, respectively, and thus get:

$$\begin{aligned} \left( { \sum \limits_{b} \ddot{z}_{{i^{j} }}^{b} \epsilon_{j}^{b} } \right)^{2} & = \sum \limits_{b} \sum \limits_{{b^{\prime}}} \ddot{z}_{{i^{j} }}^{b} \ddot{z}_{{i^{j} }}^{{b^{\prime}}} \epsilon_{j}^{b} \epsilon_{j}^{{b^{\prime}}} \\ &= \sum \limits_{{b \ne b^{*} }} \sum \limits_{{b^{\prime} \ne b^{*} }} \ddot{z}_{{i^{j} }}^{b} \ddot{z}_{{i^{j} }}^{{b^{\prime}}} \left( {\epsilon_{j}^{{b^{*} }} + \epsilon_{j}^{b} - \epsilon_{j}^{{b^{*} }} } \right)\left( {\epsilon_{j}^{{b^{*} }} + \epsilon_{j}^{{b^{\prime}}} - \epsilon_{j}^{{b^{*} }} } \right) \\ & \quad + 2 \sum \limits_{{b \ne b^{*} }} \ddot{z}_{{i^{j} }}^{b} \ddot{z}_{{i^{j} }}^{b^{*}} \epsilon_{j}^{b} \epsilon_{j}^{{b^{*} }} + {\ddot{z}_{{i^{j} }}^{{b^{*^2}}}} {\epsilon_{j}^{b^{*^2}}} . \\ \end{aligned}$$

Since:

35 $$\left( {\epsilon_{j}^{{b^{*} }} + \epsilon_{j}^{b} - \epsilon_{j}^{{b^{*} }} } \right)\left( {\epsilon_{j}^{{b^{*} }} + \epsilon_{j}^{{b^{\prime}}} - \epsilon_{j}^{{b^{*} }} } \right) = - {\epsilon_{j}^{b^{*}}}^2 + \epsilon_{j}^{b} \epsilon_{j}^{{b^{*} }} + \epsilon_{j}^{{b^{\prime}}} \epsilon_{j}^{{b^{*} }} + \left( {\epsilon_{j}^{{b^{\prime}}} - \epsilon_{j}^{{b^{*} }} } \right)\left( {\epsilon_{j}^{b} - \epsilon_{j}^{{b^{*} }} } \right),$$

Then:

$$\begin{aligned} \left( { \sum \limits_{b} \ddot{z}_{{i^{j} }}^{b} \epsilon_{j}^{b} } \right)^{2} & = \sum \limits_{{b \ne b^{*} }} \sum \limits_{{b^{\prime} \ne b^{*} }} \ddot{z}_{{i^{j} }}^{b} \ddot{z}_{{i^{j} }}^{{b^{\prime}}} \left( {\epsilon_{j}^{{b^{\prime}}} - \epsilon_{j}^{{b^{*} }} } \right)\left( {\epsilon_{j}^{b} - \epsilon_{j}^{{b^{*} }} } \right) - \sum \limits_{{b \ne b^{*} }} \sum \limits_{{b^{\prime} \ne b^{*} }} \ddot{z}_{{i^{j} }}^{b} \ddot{z}_{{i^{j} }}^{{b^{\prime}}} {\epsilon_{j}^{b^{*}}}^2 \\ & \quad + 2 \sum \limits_{{b \ne b^{*} }} \sum \limits_{{b^{\prime} \ne b^{*} }} \ddot{z}_{{i^{j} }}^{b} \ddot{z}_{{i^{j} }}^{{b^{\prime}}} \epsilon_{j}^{{b^{\prime}}} \epsilon_{j}^{{b^{*} }} + 2 \sum \limits_{{b \ne b^{*} }} \ddot{z}_{{i^{j} }}^{b} \ddot{z}_{{i^{j} }}^{{b^{*}}} \epsilon_{j}^{b} \epsilon_{j}^{{b^{*}}} + {\ddot{z}_{{i^{j} }}^{b^{*}}}{^{2}} {\epsilon_{j}^{b^{*}}}^2 . \\ \end{aligned}$$

Using that $\sum_{b\ne {b}^{*}}{\ddot{z}}_{{i}^{j}}^{b}=-{\ddot{z}}_{{i}^{j}}^{{b}^{*}}$ and $\sum_{{b}^{{\prime}}\ne {b}^{*}}{\ddot{z}}_{{i}^{j}}^{{b}^{{\prime}}}=-{\ddot{z}}_{{i}^{j}}^{{b}^{*}}$ on the second term, we see that the second and the fifth terms cancel out, and using that $\sum_{b\ne {b}^{*}}{\ddot{z}}_{{i}^{j}}^{b}=-{\ddot{z}}_{{i}^{j}}^{{b}^{*}}$ on the third term, we see that the third and the fourth terms cancel out. Therefore, ${\left(\sum_{b}{{\ddot{z}}_{{i}^{j}}^{b}\epsilon }_{j}^{b}\right)}^{2}=\sum_{b\ne {b}^{*}}\sum_{{b}^{{\prime}}\ne {b}^{*}}{\ddot{z}}_{{i}^{j}}^{b}{\ddot{z}}_{{i}^{j}}^{{b}^{{\prime}}}\left({\epsilon }_{j}^{b}-{\epsilon }_{j}^{{b}^{*}}\right)\left({\epsilon }_{j}^{{b}^{{\prime}}}-{\epsilon }_{j}^{{b}^{*}}\right)$, and finally, $Var\left({\ddot{\kappa }}_{{i}^{j}}^{sum}\right)=\sum_{b\ne {b}^{*}}\sum_{{b}^{{\prime}}\ne {b}^{*}}{\ddot{z}}_{{i}^{j}}^{b}{\ddot{z}}_{{i}^{j}}^{{b}^{{\prime}}}E\left[\left({\epsilon }_{j}^{b}-{\epsilon }_{j}^{{b}^{*}}\right)\left({\epsilon }_{j}^{{b}^{{\prime}}}-{\epsilon }_{j}^{{b}^{*}}\right)\right]$, which is Eq. (11).

Equation (12) about the covariance between the LBP effects of individuals $i$ and ${i}^{{\prime}}$ over all breeds $b$ for locus $j$ is derived similarly to the above derivation of Eq. (11), and we obtain:

$$Cov\left({\ddot{\kappa }}_{{i}^{j}}^{sum},{\ddot{\kappa }}_{{{i}^{{\prime}}}^{j}}^{sum}\right)=\sum_{b\ne {b}^{*}}\sum_{{b}^{{\prime}}\ne {b}^{*}}{\ddot{z}}_{{i}^{j}}^{b}{\ddot{z}}_{{{i}^{{\prime}}}^{j}}^{{b}^{{\prime}}}E\left[\left({\epsilon }_{j}^{b}-{\epsilon }_{j}^{{b}^{*}}\right)\left({\epsilon }_{j}^{{b}^{{\prime}}}-{\epsilon }_{j}^{{b}^{*}}\right)\right].$$

## Appendix 4

Here, we derive Eqs. (22) and (23), with the expectation of pedigree-based LBP variance and covariance for single loci, respectively. In a pedigree-based LBP model, we have the expectation of LBP variance as:

$$\begin{aligned} E\left[ {{\text{Var}}\left( {\kappa_{{s_{i}^{j} }}^{sum} } \right)} \right] & = \sum \limits_{b} f_{{s_{i} }}^{b} {\epsilon_{j}^{b}}^{2} - \left( { \sum \limits_{b} f_{{s_{i} }}^{b} \epsilon_{j}^{b} } \right)^{2} \\ & = \sum \limits_{b} f_{{s_{i} }}^{b} {\epsilon_{j}^{b}}^{2} - \sum \limits_{b} \sum \limits_{{b^{\prime}}} f_{{s_{i} }}^{b} f_{{s_{i} }}^{{b^{\prime}}} \epsilon_{j}^{b} \epsilon_{j}^{{b^{\prime}}} . \\ \end{aligned}$$

We set one breed as reference breed, ${b}^{*}$. Then for breeds $b\ne {b}^{*}$ and ${b}^{{\prime}}\ne {b}^{*}$, we do the substitutions ${\epsilon }_{j}^{b}={\epsilon }_{j}^{{b}^{*}}+{\epsilon }_{j}^{b}-{\epsilon }_{j}^{{b}^{*}}$, and ${\epsilon }_{j}^{{b}^{{\prime}}}={\epsilon }_{j}^{{b}^{*}}+{\epsilon }_{j}^{{b}^{{\prime}}}-{\epsilon }_{j}^{{b}^{*}}$, and we get:

$$\begin{aligned} E\left[ {{\text{Var}}\left( {\kappa_{{s_{i}^{j} }}^{sum} } \right)} \right] & = \sum \limits_{{b \ne b^{*} }} f_{{s_{i} }}^{b} \left( {\epsilon_{j}^{{b^{*} }} + \epsilon_{j}^{b} - \epsilon_{j}^{{b^{*} }} } \right)^{2} + f_{{s_{i} }}^{{b^{*} }} {\epsilon_{j}^{b^{*}}}^2 \\ & \quad - \sum \limits_{{b \ne b^{*} }} \sum \limits_{{b^{\prime} \ne b^{*} }} f_{{s_{i} }}^{b} f_{{s_{i} }}^{{b^{\prime}}} \left( {\epsilon_{j}^{{b^{*} }} + \epsilon_{j}^{b} - \epsilon_{j}^{{b^{*} }} } \right)\left( {\epsilon_{j}^{{b^{*} }} + \epsilon_{j}^{{b^{\prime}}} - \epsilon_{j}^{{b^{*} }} } \right) \\ & \quad - 2 \sum \limits_{{b \ne b^{*} }} f_{{s_{i} }}^{b} f_{{s_{i} }}^{{b^{*} }} \epsilon_{j}^{b} \epsilon_{j}^{{b^{*} }} - {f_{{s_{i} }}^{b^{*}}}^2 {\epsilon_{j}^{b^{*}}}^2 . \\ \end{aligned}$$

Using Eq. (35), then:

$$\begin{aligned} E\left[ {{\text{Var}}\left( {\kappa_{{s_{i}^{j} }}^{sum} } \right)} \right] & = 2 \sum \limits_{{b \ne b^{*} }} f_{{s_{i} }}^{b} \epsilon_{j}^{b} \epsilon_{j}^{{b^{*} }} - \sum \limits_{{b \ne b^{*} }} f_{{s_{i} }}^{b} {\epsilon_{j}^{b^{*}}}^2 \\ & \quad + \sum \limits_{{b \ne b^{*} }} \left[ {f_{{s_{i} }}^{b} \left( {\epsilon_{j}^{b} - \epsilon_{j}^{{b^{*} }} } \right)^{2} } \right] + f_{{s_{i} }}^{{b^{*} }} {\epsilon_{j}^{b^{*}}}^2 + \sum \limits_{{b \ne b^{*} }} \sum \limits_{{b^{\prime} \ne b^{*} }} f_{{s_{i} }}^{b} f_{{s_{i} }}^{{b^{\prime}}} {\epsilon_{j}^{b^{*}}}^2 \\ & \quad - 2 \sum \limits_{{b \ne b^{*} }} \sum \limits_{{b^{\prime} \ne b^{*} }} f_{{s_{i} }}^{b} f_{{s_{i} }}^{{b^{\prime}}} \epsilon_{j}^{b} \epsilon_{j}^{{b^{*} }} - \sum \limits_{{b \ne b^{*} }} \sum \limits_{{b^{\prime} \ne b^{*} }} \left[ {f_{{s_{i} }}^{b} f_{{s_{i} }}^{{b^{\prime}}} \left( {\epsilon_{j}^{{b^{\prime}}} - \epsilon_{j}^{{b^{*} }} } \right)\left( {\epsilon_{j}^{b} - \epsilon_{j}^{{b^{*} }} } \right)} \right] \\ &\quad - 2 \sum \limits_{{b \ne b^{*} }} f_{{s_{i} }}^{b} f_{{s_{i} }}^{{b^{*} }} \epsilon_{j}^{b} \epsilon_{j}^{{b^{*} }} - {f_{{s_{i} }}^{b^{*}}}^2 {\epsilon_{j}^{b^{*}}}^2 . \\ \end{aligned}$$

Using that $\sum_{b\ne {b}^{*}}{f}_{{s}_{i}}^{b}=1-{f}_{{s}_{i}}^{{b}^{*}}$ on the fifth, sixth and last terms, we have:

$$\begin{aligned} E\left[ {{\text{Var}}\left( {\kappa_{{s_{i}^{j} }}^{sum} } \right)} \right] & = 2 \sum \limits_{{b \ne b^{*} }} f_{{s_{i} }}^{b} \epsilon_{j}^{b} \epsilon_{j}^{{b^{*} }} - \sum \limits_{{b \ne b^{*} }} f_{{s_{i} }}^{b} {\epsilon_{j}^{b^{*}}}^2 \\ & \quad + \sum \limits_{{b \ne b^{*} }} \left[ {f_{{s_{i} }}^{b} \left( {\epsilon_{j}^{b} - \epsilon_{j}^{{b^{*} }} } \right)^{2} } \right] + f_{{s_{i} }}^{{b^{*} }} {\epsilon_{j}^{b^{*}}}^2 + \sum \limits_{{b \ne b^{*} }} f_{{s_{i} }}^{b} {\epsilon_{j}^{b^{*}}}^2 \\ & \quad - \sum \limits_{{b \ne b^{*} }} f_{{s_{i} }}^{b} f_{{s_{i} }}^{{b^{*} }} {\epsilon_{j}^{b^{*}}}^2 - 2 \sum \limits_{{b \ne b^{*} }} f_{{s_{i} }}^{b} \epsilon_{j}^{b} \epsilon_{j}^{{b^{*} }} + 2 \sum \limits_{{b \ne b^{*} }} f_{{s_{i} }}^{b} f_{{s_{i} }}^{{b^{*} }} \epsilon_{j}^{b} \epsilon_{j}^{{b^{*} }} \\ & \quad - \sum \limits_{{b \ne b^{*} }} \sum \limits_{{b^{\prime} \ne b^{*} }} \left[ {f_{{s_{i} }}^{b} f_{{s_{i} }}^{{b^{\prime}}} \left( {\epsilon_{j}^{{b^{\prime}}} - \epsilon_{j}^{{b^{*} }} } \right)\left( {\epsilon_{j}^{b} - \epsilon_{j}^{{b^{*} }} } \right)} \right] \\ & \quad - 2 \sum \limits_{{b \ne b^{*} }} f_{{s_{i} }}^{b} f_{{s_{i} }}^{{b^{*} }} \epsilon_{j}^{b} \epsilon_{j}^{{b^{*} }} - f_{{s_{i} }}^{{b^{*} }} {\epsilon_{j}^{b^{*}}}^2 + \sum \limits_{{b \ne b^{*} }} f_{{s_{i} }}^{b} f_{{s_{i} }}^{{b^{*} }} {\epsilon_{j}^{b^{*}}}^2 . \\ \end{aligned}$$

Removing the terms that cancel out,

$$E\left[ {{\text{Var}}\left( {\kappa_{{s_{i}^{j} }}^{sum} } \right)} \right] = \sum \limits_{{b \ne b^{*} }} \left[ {f_{{s_{i} }}^{b} \left( {\epsilon_{j}^{b} - \epsilon_{j}^{{b^{*} }} } \right)^{2} } \right] - \sum \limits_{{b \ne b^{*} }} \sum \limits_{{b^{\prime} \ne b^{*} }} \left[ {f_{{s_{i} }}^{b} f_{{s_{i} }}^{{b^{\prime}}} \left( {\epsilon_{j}^{{b^{\prime}}} - \epsilon_{j}^{{b^{*} }} } \right)\left( {\epsilon_{j}^{b} - \epsilon_{j}^{{b^{*} }} } \right)} \right].$$

Therefore, we have ${n}_{b}-1$ LBP variance terms (for $b={b}^{{\prime}}$),

$$\sum \limits_{{b \ne b^{*} }} \left[ {f_{{s_{i} }}^{b} \left( {\epsilon_{j}^{b} - \epsilon_{j}^{{b^{*} }} } \right)^{2} } \right] - \sum \limits_{{b \ne b^{*} }} \left[{{f_{{s_{i} }}^{b}}^2 \left( {\epsilon_{j}^{b} - \epsilon_{j}^{{b^{*} }} } \right)^{2} } \right] = \sum \limits_{{b \ne b^{*} }} \left[ {f_{{s_{i} }}^{b} \left( {1 - f_{{s_{i} }}^{b} } \right)\left( {\epsilon_{j}^{b} - \epsilon_{j}^{{b^{*} }} } \right)^{2} } \right],$$

and ${({n}_{b}-1)}^{2}$ covariances between breeds $b$ and ${b}^{{\prime}}$,

$$\sum_{b\ne {b}^{*}}\sum_{{b}^{{\prime}}\ne \left\{b,{b}^{*}\right\}}\left[-{f}_{{s}_{i}}^{b}{f}_{{s}_{i}}^{{b}^{{\prime}}}\left({\epsilon }_{j}^{{b}^{{\prime}}}-{\epsilon }_{j}^{{b}^{*}}\right)\left({\epsilon }_{j}^{b}-{\epsilon }_{j}^{{b}^{*}}\right)\right].$$

## Appendix 5

Here, we show the relationship between BS variances and LBP variances in Eq. (32) for the variance of BS between breeds $b$ and ${b}^{{\prime}}$ when neither of the breeds is the reference breed, ${b}^{*}$. The BS variance is ${\sigma }_{w\left(b,{b}^{{\prime}}\right)}^{2}=\frac{1}{2}\sum_{j=1}^{m}{\left({\epsilon }_{j}^{b}-{\epsilon }_{j}^{{b}^{{\prime}}}\right)}^{2}$. Substituting ${\epsilon }_{j}^{b}-{\epsilon }_{j}^{{b}^{{\prime}}}\text{with}\;{(\epsilon }_{j}^{b}-{\epsilon }_{j}^{{b}^{*}})-({\epsilon }_{j}^{{b}^{{\prime}}}-{\epsilon }_{j}^{{b}^{*}})$ and rearranging gives:

$$\begin{aligned} \sigma_{{w\left( {b,b^{\prime}} \right)}}^{2} & = \frac{1}{2} \sum \limits_{j = 1}^{m} \left( {\epsilon_{j}^{b} - \epsilon_{j}^{{b^{*} }} } \right)^{2} + \frac{1}{2} \sum \limits_{j = 1}^{m} \left( {\epsilon_{j}^{{b^{\prime}}} - \epsilon_{j}^{{b^{*} }} } \right)^{2} \\ & \quad - \sum \limits_{j = 1}^{m} \left( {\epsilon_{j}^{b} - \epsilon_{j}^{{b^{*} }} } \right)\left( {\epsilon_{j}^{{b^{\prime}}} - \epsilon_{j}^{{b^{*} }} } \right) = \frac{1}{2}\sigma_{{k\left( {bb} \right)}} + \frac{1}{2}\sigma_{{k\left( {b^{\prime}b^{\prime}} \right)}} - \sigma_{{k\left( {bb^{{\prime}} } \right)}} \\ \end{aligned}$$

Furthermore, here we derive how the covariance between LBP effects of breeds $b$ and ${b}^{{\prime}}$ can be calculated from BS variance components. We have the covariance between LBP effects across breeds: ${\sigma }_{k({bb}^{{\prime}})}=\sum_{j=1}^{m}\left({\epsilon }_{j}^{b}-{\epsilon }_{j}^{{b}^{*}}\right)\left({\epsilon }_{j}^{{b}^{{\prime}}}-{\epsilon }_{j}^{{b}^{*}}\right)$. Rearranging gives:

$$\begin{aligned} \sigma_{{k\left( {bb^{{\prime}} } \right)}} &= \sum \limits_{j = 1}^{m} \left( {\epsilon_{j}^{b} \epsilon_{j}^{{b^{\prime}}} - \epsilon_{j}^{b} \epsilon_{j}^{{b^{*} }} - \epsilon_{j}^{{b^{\prime}}} \epsilon_{j}^{{b^{*} }} + {\epsilon_{j}^{b^{*}}}^2 + \frac{1}{2} {\epsilon_{j}^{b}}^2 + \frac{1}{2} {\epsilon_{j}^{b^{\prime}}}^2 - \frac{1}{2} {\epsilon_{j}^{b}}^2 - \frac{1}{2} {\epsilon_{j}^{b^{\prime}}}^2 } \right) \\ & = \frac{1}{2} \sum \limits_{j = 1}^{m} \left( {\epsilon_{j}^{b} - \epsilon_{j}^{{b^{*} }} } \right)^{2} + \frac{1}{2} \sum \limits_{j = 1}^{m} \left( {\epsilon_{j}^{{b^{\prime}}} - \epsilon_{j}^{{b^{*} }} } \right)^{2} - \frac{1}{2} \sum \limits_{j = 1}^{m} \left( {\epsilon_{j}^{b} - \epsilon_{j}^{{b^{\prime}}} } \right)^{2} . \\ \end{aligned}$$

Therefore,

$${\sigma }_{k({bb}^{{\prime}})}={\sigma }_{w\left(b,{b}^{*}\right)}^{2}+{\sigma }_{w\left({b}^{{\prime}},{b}^{*}\right)}^{2}-{\sigma }_{w\left(b,{b}^{{\prime}}\right)}^{2}.$$
